# PREDICTOR: A Non‐Enzymatic Catalytic Cascade Tool for in Situ Visualization of Small Extracellular Vesicle Surface glycoRNAs

**DOI:** 10.1002/jev2.70282

**Published:** 2026-04-14

**Authors:** Shuang Xie, Ben Niu, Ruijia Deng, Liu Feng, Zuowei Xie, Shuang Zhao, Hongzhao Yang, Meilin Gong, Jing Sheng, Ligai Zhang, Yan Pi, Ningtao Cheng, Ming Chen, Kai Chang

**Affiliations:** ^1^ Department of Clinical Laboratory Medicine Southwest Hospital, Third Military Medical University (Army Medical University) Chongqing China; ^2^ Department of Rehabilitation Medicine The First Affiliated Hospital of Chongqing Medical University Chongqing China; ^3^ School of Public Health Zhejiang University School of Medicine Hangzhou Zhejiang China; ^4^ State Key Laboratory of Trauma and Chemical Poisoning Army Medical University Chongqing China

**Keywords:** DNA nanotechnology, glycoRNAs, in situ visualization, non‐enzymatic cascade amplification, small extracellular vesicles

## Abstract

Glycosylated RNAs (glycoRNAs) are membrane‐displayed RNA‐glycan conjugates, but quantitative in situ analysis of low‐abundance glycoRNAs on small extracellular vesicles (sEVs) remains challenging. Here, we develop PREDICTOR (proximity‐encoded non‐linear hybridization chain reaction circuit), an enzyme‐free catalytic DNA cascade for imaging and quantifying surface glycoRNAs on intact sEVs. PREDICTOR uses a sialic acid aptamer and an RNA‐sequence probe as split recognition modules, whose proximity on a single glycoRNA reconstitutes an initiator that drives dendritic DNA self‐assembly and non‐linear fluorescence amplification on the sEV surface. Across serial sEV inputs (5×10^3^–5×10^9^ particles·mL^−^^1^), PREDICTOR exhibited a steeper concentration‐response than representative amplification assays, with higher regression slopes in bulk fluorescence (0.372, *R* ^2^ = 0.9846) compared with ARPLA (0.301, *R* ^2^ = 0.9748) and HieCo 2 (0.230, R^2^ = 0.9663), and similarly improved performance in bead‐based imaging (4.105, *R* ^2^ = 0.9816 vs. 3.495 and 2.711). Applying PREDICTOR to a breast cancer transformation model (MCF‐10A → MCF‐7 → MDA‐MB‐231) showed that multiple surface glycoRNA candidates decrease with malignancy, accompanied by progressive softening of sEV membranes (Young's modulus reduced by 25.08% and 54.72%). Functionally, enzymatic depletion of surface RNA and disruption of sialylated N‐glycan features attenuated macrophage uptake and inflammatory activation, supporting a contribution of surface glycoRNAs to sEV recognition. Collectively, PREDICTOR provides a rapid, enzyme‐free platform for quantitative and spatial profiling of sEV surface glycoRNAs and links their abundance to vesicle mechanics and immune‐cell interactions.

## Graphical abstract

PREDICTOR, a non‐enzymatic catalytic circuit for imaging trace glycoRNAs on sEVs, reveals that glycoRNAs abundance negatively correlates with tumour malignancy and is functionally important for sEVs uptake by macrophages

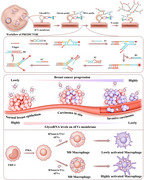

## 1 Introduction

Glycosylated RNAs (glycoRNAs) were recently discovered as a class of RNA‐glycan conjugates in which small non‐coding RNAs carry predominantly sialylated and fucosylated N‐glycans and are presented on the extracellular face of mammalian cell membranes (Flynn et al. 2021; Jiang et al. 2025; Sharma et al. 2025). Mechanistic studies are beginning to clarify how these RNA‐glycan conjugates are generated and displayed, including the identification of RNA nucleoside attachment sites for N‐glycans (Xie et al. 2024; Yadav et al. 2026). Functionally, cell‐surface glycoRNAs can engage sialic acid‐binding immunoglobulin‐like lectins (Siglecs) and participate in immune regulation, placing RNA glycosylation at the interface of glycobiology and cell communication (Disney 2021; Graziano et al. 2025; Montag et al. 2025; Zhang et al. 2024). Recent reviews have summarized advances in glycoRNA biogenesis, detection methods, and disease relevance (Aquino‐Jarquin 2026; Yi et al. 2025; Zhang et al. 2025).

Small extracellular vesicles (sEVs) are nano‐sized lipid‐bilayer particles that disseminate proteins, lipids, and nucleic acids and are increasingly investigated as mediators and biomarkers of disease (Abhange et al. 2021; Möller and Lobb 2020; Zhou et al. 2024). Beyond luminal RNA cargo, sEV membranes present a dense array of glycoconjugates that shape vesicle stability, biodistribution, and interactions with recipient cells (Rohm et al. 2025). Recent reports indicate that glyco‑modified RNAs can be associated with extracellular vesicles both within the vesicle lumen and on the vesicle surface, raising the possibility that glycoRNAs contribute to vesicle recognition and uptake in trans (Ren et al. 2025; Sharma et al. 2025).

Deciphering sEV‑associated glycoRNAs requires methods that are sensitive at low vesicle inputs, compatible with intact membranes, and able to discriminate RNA‑linked glycans from the overwhelming background of canonical glycoproteins and glycolipids. Existing approaches‐including metabolic labelling coupled to enrichment/sequencing, lectin/antibody staining, and in situ imaging have provided important first insights but remain technically demanding for routine, quantitative sEV profiling. For example, amplification‑assisted proximity ligation approaches (ARPLA) rely on enzyme‑mediated ligation and rolling‑circle amplification, whereas FRET‑based strategies (drFRET) provide enzyme‑free readouts but without signal gain and typically require carefully optimized donor/acceptor labelling (Ma et al. 2024; Ren et al. 2025). More recently, programmable DNA‑circuit imaging methods (e.g., HieCo2), as well as intramolecular proximity‑induced amplification (IPIA), have expanded the in situ toolbox by leveraging nucleic‑acid reaction networks (Gong et al. 2025; Liu et al. 2024). Nevertheless, further improvements in operational simplicity, background suppression, and quantitative performance are still needed particularly for vesicle‑surface targets that can be present at low copy number.

Here we introduce PREDICTOR, an enzyme‑free, proximity‑encoded catalytic DNA cascade for in situ visualization and quantification of surface glycoRNAs on sEVs. PREDICTOR uses two split probes: a Neu5Ac‑binding aptamer to report sialylated glycan features and a sequence‑specific RNA probe. Only when both probes co‑localize on a single glycoRNA is an initiator reconstituted to trigger a non‑linear hybridization chain reaction (HCR) cascade that assembles dendritic DNA nanostructures directly on the sEV membrane, yielding strong fluorescence amplification. We apply PREDICTOR to profile sEV surface glycoRNAs in a breast cancer cell line model, benchmark its quantitative performance against representative state‑of‑the‑art amplification workflows, and explore functional links between surface glycoRNAs, vesicle mechanics, and macrophage uptake/internalization.

## 2 Materials and Methods

### 2.1 Cell Culture

The human breast cancer cell lines MCF‐7 and MDA‐MB‐231, the human normal breast epithelial cell line MCF‐10A, and the human monocytic cell line THP‐1 were purchased from Pricells (Wuhan, China). MCF‐7 and MDA‐MB‐231 cells were cultured in DMEM (Thermo Fisher Scientific) supplemented with 10% foetal bovine serum (FBS) and 1% penicillin‐streptomycin at 37°C in a humidified incubator with 5% CO_2_. MCF‐10A cells were cultured in complete medium (CM‐0525, Procell) under the same conditions. THP‐1 cells were cultured in RPMI‐1640 (Thermo Fisher Scientific) supplemented with 10% FBS and 0.05 mM β‐mercaptoethanol at 37°C with 5% CO_2_.

### 2.2 Metabolic Labelling of the Cells

N‐azidoacetylmannosamine tetraacylated (Ac4ManNAz; MedChemExpress) was prepared as a 500 mM stock solution in sterile DMSO. N‐acetyl‐D‐galactosamine (GalNAc; Sigma) was prepared as a 500 mM stock, and D‐(+)‐galactose (Gal; Sigma) as a 50 mM stock in sterile water. For metabolic labelling, Ac4ManNAz was added to the culture medium at a final concentration of 100 µM, together with GalNAc (100 µM) and Gal (10 µM) as indicated, following an established glycoRNA metabolic‐labelling workflow (Flynn et al. 2021). Cells were incubated for 36 h unless otherwise specified.

### 2.3 sEVs Isolation

The MCF‐7, MDA‐MB‐231, and MCF‐10A cell lines were labelled as described above. Subsequently, they were washed and incubated in phenol red‐free DMEM medium not containing FBS for 48 h. Following this incubation, the phenol red‐free DMEM medium without FBS was collected, and sEVs were isolated using a serial ultracentrifugation protocol. Briefly, the supernatants were first centrifuged at 2000× g for 20 min, followed by centrifugation at 10,000× g for 50 min at 4°C. The resulting supernatants were then filtered through a 0.22 µm filter to eliminate cell debris and larger extracellular vesicles (>220 nm). sEVs were subsequently pelleted by ultracentrifugation at 100,000×g for 70 min at 4°C (Optima XE‐100, Beckman), washed with ice‐cold PBS, and pelleted again using the same method.

### 2.4 Western Blot Analysis

Total proteins were extracted from sEVs derived from the MCF‐7, MDA‐MB‐231, and MCF‐10A cell lines, with or without Ac4ManNAz treatment or macrophage cells, using radio‐immunoprecipitation assay (RIPA) cell lysis buffer supplemented with protease inhibitors. Protein concentrations were quantified using the BCA method. Cell lysates were separated via 12% sodium dodecyl sulphate‐polyacrylamide gel electrophoresis (SDS‐PAGE) and subsequently transferred to a polyvinylidene difluoride (PVDF) membrane. The membranes were blocked with 5% skim milk in Tris‐buffered saline with Tween‐20 for 2 h at room temperature (RT). Following this, the membranes were incubated overnight at 4°C with primary antibodies, including rabbit anti‐mouse/human Calnexin (1:1000), TSG101 (1:1000), CD63 (1:1000), GAPDH (1:1000), iNOS (1:1000), and β‐actin (1:1000), all diluted in 5% skim milk. The membranes were then washed twice with TBST solution for 5 min each time and incubated with horseradish peroxidase‐labelled secondary antibody (1:5000) for 1 h at room temperature. Target bands were visualized using the Enhanced ECL chemiluminescence kit, and protein bands were quantified using ImageJ software. The ratio of grey value between the target band and the internal reference band (GAPDH or β‐actin) was calculated to represent the relative expression of the target proteins. Experiments were conducted in triplicate.

### 2.5 Characterization of sEVs

For NTA measurement, sEVs labelled with or without Ac_4_ManNAz from MCF‐10A, MCF‐7, and MDA‐MB‐231 cells were diluted in PBS to a final volume of 1 mL, achieving a concentration of approximately 10^7^–10^9^ particles mL^−1^. A sample was loaded into the Nanosight chamber using a syringe, ensuring that no bubbles were introduced. The settings were configured according to the manufacturer's software manual (NanoSight NS300 User Manual, MAN0541‐01‐EN‐00, 2017). For transmission electron microscopy (TEM) analysis, a 10 µL drop of the sample was applied to a carbon‐coated grid that had been glow discharged for 1 min in air. The grids were then immediately negatively stained using 2% phosphotungstic acid (Rhawn) for 60 s. Subsequently, the grids were examined using an H‐7800 microscope (HITACHI) operated at 80–120 kV.

### 2.6 RNA Extraction and Purification

TRIzol (Thermo Fisher Scientific) was utilized to lyse and denature the sEVs. The samples were homogenized in TRIzol by pipetting up and down and incubated at 37°C for 15 min. To separate the phases, 0.2×volume of 100% chloroform was added, and the mixture was vortexed before being centrifuged at 12,000×g for 15 min at 4°C. The aqueous phase was then carefully removed and transferred to a fresh tube, where it was mixed with 2×volumes of 100% ethanol. The RNA pellet was subsequently dissolved in UltraPure DNase/RNase‐Free Distilled Water. For additional purification, Proteinase K (Thermo Fisher Scientific) was added to purified RNA to a final concentration of 1 mg/mL in 11.1 mM Tris‐HCl (pH 7.5) and incubated for 30 min at 37°C for protein digestion. After digestion, RNA was purified using a Zymo RNA Clean and Concentrator column (Zymo Research) according to the manufacturer's protocol. All RNA samples extracted in this study underwent these purification steps prior to any subsequent enzymatic treatment or biotin labelling, unless specified otherwise.

### 2.7 Enzymatic Treatment of RNA Samples and sEVs

Enzymatic treatments of RNA samples and sEVs were performed as indicated. For sialidase digestion, α2‐3,6,8,9 neuraminidase A (20 U/µL; New England Biolabs) was used in 1× GlycoBuffer 1 at 37°C for 2 h. For N‐glycan removal, PNGase F (500,000 U/mL; New England Biolabs) was used in 1× GlycoBuffer 2 at 37°C for 2 h. For O‐glycan removal, O‐glycosidase (40,000 U/mL; New England Biolabs) was used in 1× GlycoBuffer 2 at 37°C for 2 h. For defucosylation, α1‐2,3,4,6 fucosidase (10 U/µL; Sigma–Aldrich) was used in 50 mM sodium acetate (pH 5.0) at 37°C for 2 h. For protein digestion, proteinase K (800 U/mL) was used in PBS at 37°C for 2 h. For DNA removal, samples were treated with DNase I (final 100 U/mL; Roche) at 37°C for 60 min, followed by RNA purification using a Zymo RNA Clean and Concentrator column (Zymo Research). For RNA digestion, a mixture of RNase A and RNase T1 (final 200 U/mL and 5000 U/mL, respectively) was used under non‐permeabilizing, detergent‐free conditions (RNase‐free PBS) at 37°C (30 min unless otherwise specified). Where indicated, RNase activity was inhibited by pre‐incubating RNase inhibitor (10 U/µL; Roche) with the RNase mixture at 25°C for 30 min prior to addition. Unless otherwise stated, untreated controls were processed in parallel using the corresponding buffers without enzymes. After enzymatic treatments, RNA was purified for downstream analyses; for intact sEV digestions, residual enzymes were removed by washing/pelleting as needed prior to subsequent labelling or uptake assays.

### 2.8 Copper‐Free Click Chemistry Reaction

For copper‐free click chemistry, azide‐labelled RNA (or azide‐labelled sEV preparations) generated by Ac_4_ManNAz metabolic labelling was conjugated to biotin using DBCO‐PEG_4_‐biotin (Sigma–Aldrich). Purified RNA samples were incubated with DBCO‐PEG_4_‐biotin (final 500 µM) at room temperature for 2 h, and then purified by ethanol precipitation or column cleanup. For sEV labelling, sEV suspensions were incubated with DBCO‐PEG4‐biotin (final 500 µM) with gentle mixing, followed by removal of excess reagent by washing/pelleting prior to downstream capture or imaging.

### 2.9 RNA Gel Electrophoresis, Blotting, and Imaging

Biotin‐labelled RNA was analysed by a Northern blot‐like workflow with minor modifications. Purified, enriched, or enzymatically treated RNA samples were mixed with dye‐free gel loading buffer II (df‐GLBII; Thermo Fisher Scientific) supplemented with SYBR Gold (Invitrogen) and DBCO‐PEG4‐biotin (final 500 µM), incubated at 50°C for 5 min, and then purified by ethanol precipitation (2.5 volumes of ethanol and 0.1 volume of 3 M sodium acetate, pH 5.2). RNA pellets were washed with 75% ethanol, air‐dried, and resuspended in RNase‐free water. Samples were resolved on a 1% denaturing agarose gel containing 2.2 M formaldehyde. The gel was imaged (SYBR Gold channel) and then transferred to a nitrocellulose membrane using a Northern blot apparatus. Membranes were UV‐crosslinked (120 mJ/cm^2^, 254 nm), blocked in 1× PBST containing 5% (w/v) BSA for 1 h, and incubated with streptavidin‐HRP (1:1000 in PBST + 5% BSA) for 30 min at room temperature. After washing (3×, 5 min each), signals were developed using an ECL substrate (Vazyme) and imaged on a chemiluminescence system.

### 2.10 Design and Assembly of Panel‐PREDICTOR

First, fluorescence‐quenched duplex substrates (S1 and S2) were prepared by annealing the corresponding fluorophore‐labelled strand (F‐strand; 5′‐FAM) and quencher‐labelled strand (Q‐strand; 3′‐BHQ1) (Table S1). Briefly, F‐and Q‐strands were mixed at a molar ratio of 1:1.5, heated to 95°C for 5 min, and cooled to room temperature over 15 min. For in vitro fluorescence assays, the PREDICTOR circuit (S1, S2, A1, A2, and Trigger) was assembled at a molar ratio of 2:2:2:4:1. For in situ imaging experiments, S1, S2, A1, A2, glycan probes, and RNA probes were assembled at a molar ratio of 2:2:2:4:1:1 in double recognition buffer (50 mM Tris‐HCl, 5 mM KCl, 100 mM NaCl, and 1 mM MgCl2, pH 7.4). The formation of the PREDICTOR circuit was verified using 12% native PAGE. In addition, dynamic DNA nanostructures generated by the PREDICTOR cascade were imaged using fluidic‐mode AFM (NanoWizard BioAFM, JPK Instruments) after deposition onto freshly cleaved mica. All oligonucleotides were synthesized and HPLC‐purified by Sangon Biotech (Shanghai, China).

### 2.11 Atomic Force Microscopy (AFM)

sEV samples were diluted to the same particle concentration, deposited on freshly cleaved mica for 10 min, and then dried with nitrogen. Images were acquired using a NanoWizard BioAFM system (JPK/Bruker), and image background was processed using Nanoscope 2.0 software.

### 2.12 In Situ Imaging of glycoRNAs on sEVs With PREDICTOR

To image glycoRNAs on sEVs by confocal microscopy, sEVs were immobilized on poly‐L‐lysine‐coated confocal dishes. Briefly, poly‐L‐lysine (0.1 mg/mL; Biosharp) was added to the dishes and incubated at 37°C for 30 min. Excess poly‐L‐lysine was removed, dishes were dried overnight at 37°C, and then washed three times with pre‐cooled PBS. Next, 1 mL of sEVs (1 × 10^6^ particles/mL) was added to each coated dish and incubated at 37°C for 2 h to allow immobilization; dishes were then washed three times with pre‐cooled PBS to remove unbound sEVs. Immobilized sEVs were divided into six groups and incubated for 1 h at 25°Cwith the indicated probe combinations prepared in double‐recognition buffer (50 mM Tris‐HCl, 5 mM KCl, 100 mM NaCl, and 1 mM MgCl_2_, pH 7.4): S1 + glycan probe; S1 + RNA probe; S1 + glycan probe + RNA probe; S1 + glycan probe + RNA probe + A1; S1 + glycan probe + RNA probe + A1 + S2; and S1 + glycan probe + RNA probe + A1 + S2 + A2 (PREDICTOR). After washing three times with pre‐cooled PBS, samples were imaged on a confocal microscope (Zeiss LSM 900, Germany).

### 2.13 Verification of Specificity of PREDICTOR

To verify the specificity of PREDICTOR, we modulated the expression levels of the glycan and RNA components of glycoRNAs and evaluated whether PREDICTOR selectively responded to the intended targets. For the RNA component, isolated sEVs were treated with RNase (A/T1) as described above. To perturb the glycan component, pharmacological and enzymatic approaches were employed. In the pharmacological approach, HeLa cells were treated with NGI‐1 (8 µM) and kifunensine (2 µM) during Ac4ManNAz labelling, and sEVs were subsequently isolated from the conditioned medium. In the enzymatic approach, isolated sEVs were treated with PNGase F (500 U·µL^−^^1^, NEB) and/or O‐glycosidase (40,000 U·µL^−^^1^, NEB) to remove the glycan portion from the surface of sEVs. For bead‐based analysis, aldehyde/sulfate latex beads were vortexed briefly and washed three times with 1 mL PBS (10,000 g, 5 min) to remove storage stabilizers. Subsequently, 200 µL PBS and 10 µL sEVs were added to the beads and incubated with gentle shaking for 2 h to allow adsorption. Beads were pelleted (10,000 g, 5 min), blocked with 1 mL 0.1 M glycine for 1 h at room temperature, and washed three times with PBS. Finally, bead‐sEV complexes were resuspended in 200 µL PBS, incubated with PREDICTOR (10 µL) for 1 h in the dark, washed three times with PBS, and analysed by confocal laser scanning microscopy (CLSM, Zeiss LSM 900, Germany) and/or flow cytometry (CytoFLEX LX, Beckman).

### 2.14 SEM

Suitable amount of sEVs‐adsorbed sEVs‐aldehyde/sulfate latex bead composites was dropped onto a silicon wafer and dried. The dried sample was then adhered to conductive adhesive and coated with gold using a Quorum SC7620 sputter coater for 45 s at a sputtering current of 10 mA. Subsequently, the sample morphology was observed using SEM (ZEISS Sigma 300) at an accelerating voltage of 3 kV.

### 2.15 Lipid Raft Staining and Imaging

To label lipid rafts on sEVs, the Vybrant Alexa Fluor 555 Lipid Raft Labelling Kit (Invitrogen) was used. Briefly, 2 µL of cholera toxin subunit B (CTB) stock solution (1 mg/mL) was added to 1 mL of HeLa‐derived sEVs (1×10^6^ particles/mL) and incubated on ice for 15 min. CTB antibody was then added and incubated on ice for 30 min. After three washes with pre‐cooled PBS, labelled sEVs were immobilized on confocal dishes, incubated with PREDICTOR for 1 h at 25°C, and imaged by confocal microscopy. Co‐localization was analysed using Fiji (ImageJ). In addition, CTB and PREDICTOR fluorescence intensities were quantified by flow cytometry to further assess co‐localization between lipid rafts and surface glycoRNA signals on sEVs.

### 2.16 sEVs Uptake Assays

THP‐1 cells were seeded into 6‐well plates at 3×10^5^ cells per well and differentiated into M0 macrophages by PMA treatment (100 nM) for 72 h. After differentiation, cells were washed and incubated for an additional 24 h in sEV‐free medium. sEVs, with or without RNase (A/T1) treatment as described above, were fluorescently labelled with PKH26 (Sigma) following the manufacturer's protocol, and excess dye was removed using Total Exosome Isolation Reagent (Invitrogen) or by repeated washing/centrifugation as indicated. Labelled sEVs (150 µg total protein per well) were added to macrophages and incubated for 6 h. After incubation, cells were washed thoroughly with PBS to remove unbound sEVs. Uptake was analysed by confocal microscopy (Zeiss LSM 900, Germany) and flow cytometry (CytoFLEX LX, Beckman).

### 2.17 Comparative Benchmarking Assays (ARPLA and HieCo2)

To benchmark PREDICTOR against representative glycoRNA signal‐amplification workflows, ARPLA and HieCo2 comparator assays were performed under matched sEVs inputs and readout settings (Figures S3 and S4). Serial dilutions of sEVs (5×10^3^–5×10^9^ particles·mL^−^^1^, quantified by NTA[nanoparticle tracking analysis]) were prepared in PBS. For bulk fluorescence benchmarking (Figure S3), total glycoRNA was extracted from each sEV input using the same RNA extraction/purification workflow described above, and equal volumes of the resulting RNA samples were subjected to PREDICTOR, ARPLA, or HieCo2 reactions in parallel. End‐point fluorescence was recorded using identical excitation/emission settings across assays, with buffer‐only and probe‐only reactions included as blanks.

ARPLA and HieCo2 were implemented by following the published protocols (Ma et al. 2024; Liu et al. 2024) with minor modifications. Briefly, ARPLA uses a sialic‐acid aptamer and an RNA‐specific probe to enable proximity ligation that forms an amplifiable circular template, followed by phi29 polymerase‐driven rolling circle amplification (RCA) and fluorescent probe hybridization for signal readout. HieCo2 uses metabolic chemical reporter labelling and a hierarchical DNA‐coding strategy to realize dual recognition of sialylated RNAs and to trigger HCR‐based amplification. In this study, the fluorescent reporter strands/probes used for signal readout were synthesized with a 5′‐FAM label to enable unified imaging/plate‐reader settings; all other oligonucleotide sequences, buffer compositions, and incubation conditions were kept consistent with the corresponding original reports. For bead‐based imaging benchmarking (Figure S4), sEVs at the indicated inputs were immobilized on aldehyde/sulfate latex beads as described above, and the bead‐sEV complexes were processed using PREDICTOR, ARPLA, or HieCo2 in parallel. After washing, beads were imaged by confocal microscopy with identical acquisition parameters, and fluorescence intensities were quantified in Fiji (ImageJ).

### 2.18 Siglec‐11 Binding Assay and Siglec‐11 Overlay Blot

For the Siglec‐11 interaction assay (Figure S8), THP‐1‐derived M0 macrophages were prepared as described above (PMA differentiation, 72 h). Cells were washed with PBS and treated with RNase (A/T1) (final 200 U·mL^−^^1^ RNase A and 5000 U·mL^−^^1^ RNase T1) or the corresponding RNase‐free buffer control for 30 min at 37°C to evaluate RNase sensitivity of surface‐accessible RNA‐dependent Siglec‐11 binding. After washing with ice‐cold FACS buffer (PBS supplemented with 2% FBS), cells were stained with recombinant human Siglec‐11 following a reported Siglec‐based staining workflow (Flynn et al. 2021) and analysed by flow cytometry (CytoFLEX LX, Beckman).

For Siglec‐11 overlay blotting (Figure S9), total RNA extracted from THP‐1‐derived M0 macrophages (with or without RNase (A/T1) treatment) was resolved and transferred to nitrocellulose as described in Section 2.9. After crosslinking, membranes were blocked with Intercept PBS Blocking Buffer (LI‐COR) (or 5% BSA in TBS‐T) at 4°C for 60 min and incubated with recombinant Siglec‐11 at 4°C (typically overnight) to allow binding to sialylated glycoRNA species, following the Siglec‐based detection strategy described previously (Flynn et al. 2021). After washing, bound Siglec‐11 was detected using an HRP‐conjugated anti‐human IgG secondary antibody (Fc‐specific) and chemiluminescence imaging on a ChemiDoc MP system.

### 2.19 Statistical Analysis

All data are presented as mean ± standard deviation (SD) from three independent experiments, unless otherwise stated. Statistical analyses were performed using GraphPad Prism 8.0. Differences between two groups were analysed using a two‐tailed unpaired Student's *t*‐test. Comparisons among more than two groups were performed using one‐way ANOVA followed by Tukey's multiple‐comparison test. *P* < 0.05 was considered statistically significant.

## 3 Results

### 3.1 glycoRNAs on the Surface of sEVs

**FIGURE 1:**
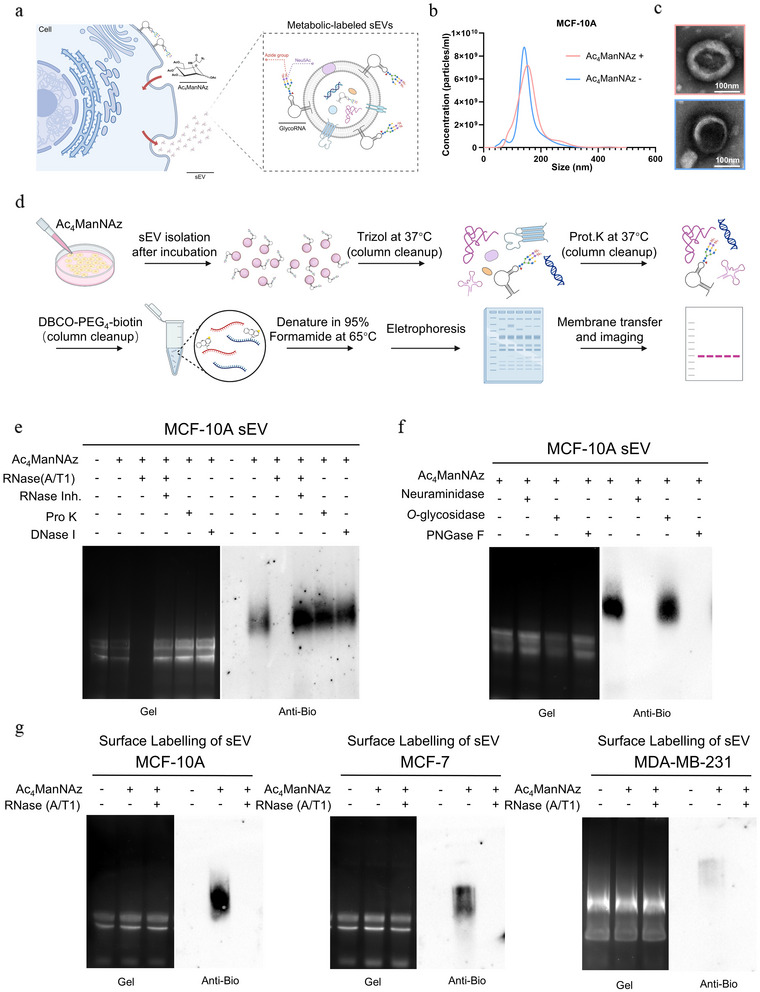
**glycoRNAs are present and surface accessible on sEVs**. (a) Metabolic labelling strategy: Ac4ManNAz enters the sialic‑acid biosynthetic pathway to install azide reporters on sialylated glycoconjugates, enabling copper‑free click labelling. (b) NTA size distribution of sEVs isolated from MCF‑10A cells (representative). (c) Representative TEM images of sEVs from untreated and Ac4ManNAz‑labeled cells. (d) Workflow for RNA extraction, click labelling with DBCO‑PEG4‑biotin, and blot detection. Pro K, proteinase K. (e) Streptavidin blot of sEV RNAs after click labelling, with the indicated treatments (RNase A/T1; RNase inhibitor; Pro K; DNase I). (f) Effect of glycosidase treatments (neuraminidase, PNGase F, or O‑glycosidase) on the biotin signal. (g) Click labelling performed directly on intact, non‑permeabilized sEVs followed by RNase A/T1 treatment of intact vesicles and RNA blotting, enabling assessment of surface‑accessible glycoRNAs. Representative images are shown.

To establish the presence of glycoRNAs associated with sEVs, we metabolically labelled breast epithelial cells (MCF‑10A, MCF‑7, and MDA‑MB‑231) with peracetylated N‑azidoacetylmannosamine (Ac4ManNAz), which is processed through the sialic‑acid biosynthetic pathway to install azide handles on sialylated glycoconjugates. After collecting conditioned medium, sEVs were isolated by ultracentrifugation and characterized by NTA, TEM, and immunoblotting for canonical sEVs markers (TSG101 and CD63) and the absence of the endoplasmic‑reticulum marker calnexin (Figures 1b and 1c; Figure S1). Total RNA isolated from labelled sEVs was reacted with DBCO‑PEG4‑biotin via copper‑free click chemistry, enabling streptavidin‑based detection (Figure 1d). A robust biotin signal was observed only in Ac4ManNAz‑labeled samples and was abolished by an RNase cocktail (RNase A/T1), whereas proteinase K or DNase I treatment had no effect (Figure 1e). In addition, the labelling signal was eliminated by neuraminidase or PNGase F, but not by O‑glycosidase (Figure 1f), consistent with sialylated, N‑linked glycoRNA structures.

To test whether these glycoRNAs are exposed on the outer surface of intact sEVs, we performed the click reaction directly on purified vesicles under non‑permeabilizing conditions, followed by RNA extraction and blotting. Strong labelling was detected and was significantly reduced when intact sEVs were pre‑treated with RNase (A/T1), indicating that a substantial fraction of the biotin‑reactive glycoRNAs is surface accessible (Figure 1g). Notably, across the breast‑cancer malignancy series, the abundance of surface‑accessible glycoRNA signal decreased from MCF‑10A to MCF‑7 to MDA‑MB‑231 sEVs, consistent with previous reports linking surface glycoRNA levels with malignant phenotypes (Ma et al. 2024).

### 3.2 Assembly and Characterization of the PREDICTOR

**FIGURE 2:**
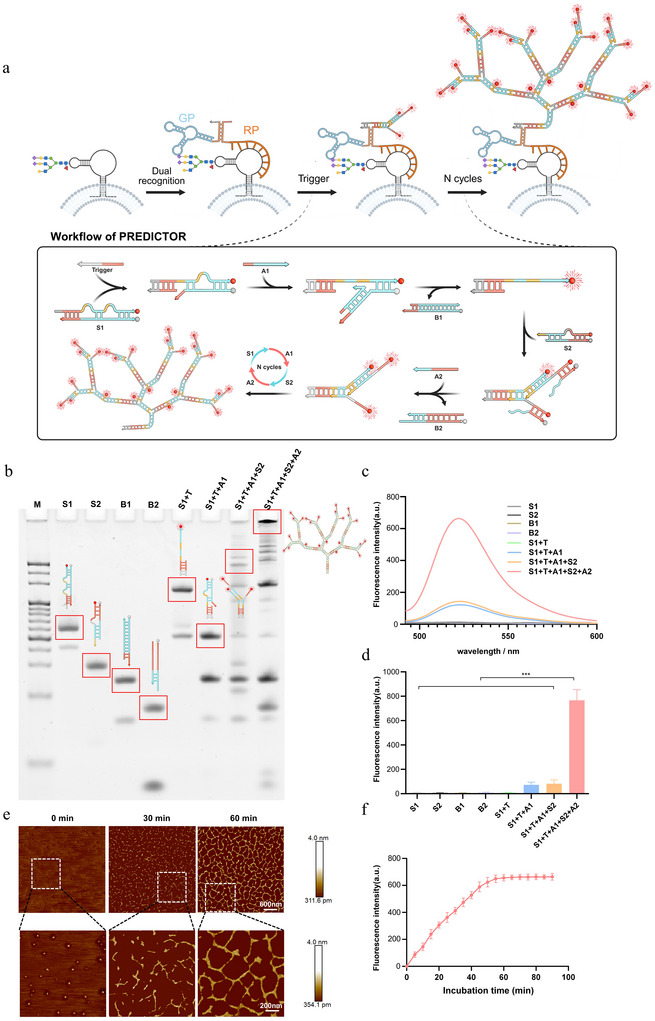
**Design and characterization of the PREDICTOR catalytic DNA cascade**. (a) Schematic of proximity‑encoded trigger reconstitution and dendritic DNA growth. (b) Native PAGE analysis of PREDICTOR self‑assembly (M, 20 bp DNA ladder). (c) Fluorescence analysis of PREDICTOR during assembly. (d) Quantification of fluorescence output during PREDICTOR self‑assembly. (e) AFM images showing branched DNA nanostructure growth at 0, 30, and 60 min. (f) Time‑dependent fluorescence intensity during assembly (*n* = 3; mean ± SD).

To enable in situ visualization of surface glycoRNAs on intact sEVs, we designed PREDICTOR, which comprises a recognition module and an assembly (signal‑amplification) module (Figure 2a). The recognition module consists of two split probes: a glycan probe (GP) and an RNA probe (RP). The GP integrates a Neu5Ac‑binding aptamer, a spacer, and a split trigger strand, allowing it to report sialylated glycan features. The RP contains a sequence‑complementary region for the target RNA together with a matching split trigger strand. Only when GP and RP bind in close proximity on a single glycoRNA does the split trigger reconstitute via hybridization, generating an initiator for downstream amplification.

The assembly module translates this proximity‑gated initiator into amplified fluorescence through an enzyme‑free, toehold‑mediated strand‑displacement cascade. Two fluorophore/quencher‑paired substrates (S1 and S2) and two auxiliary strands (A1 and A2) are designed such that the initiator sequentially opens the substrates, separates fluorophore from quencher to unmask fluorescence, and exposes new toeholds that recruit additional substrates. This catalytic process drives the in situ growth of highly branched, high‑molecular‑weight dendritic DNA nanostructures and yields non‑linear signal gain over time.

Using a synthetic trigger strand, we experimentally verified the feasibility of the designed circuit. Native PAGE showed progressive formation of higher‑order DNA assemblies when all components were present (Figure 2b). Consistent with the catalytic strand‑displacement design, fluorescence measurements revealed a time‑dependent increase in signal and a trigger‑dose‑dependent response (Figure 2c,d,f), while AFM directly visualized the growth of branched DNA architectures over time (Figure 2e).

### 3.3 PREDICTOR Identifies glycoRNAs on the Surface of sEVs

**FIGURE 3:**
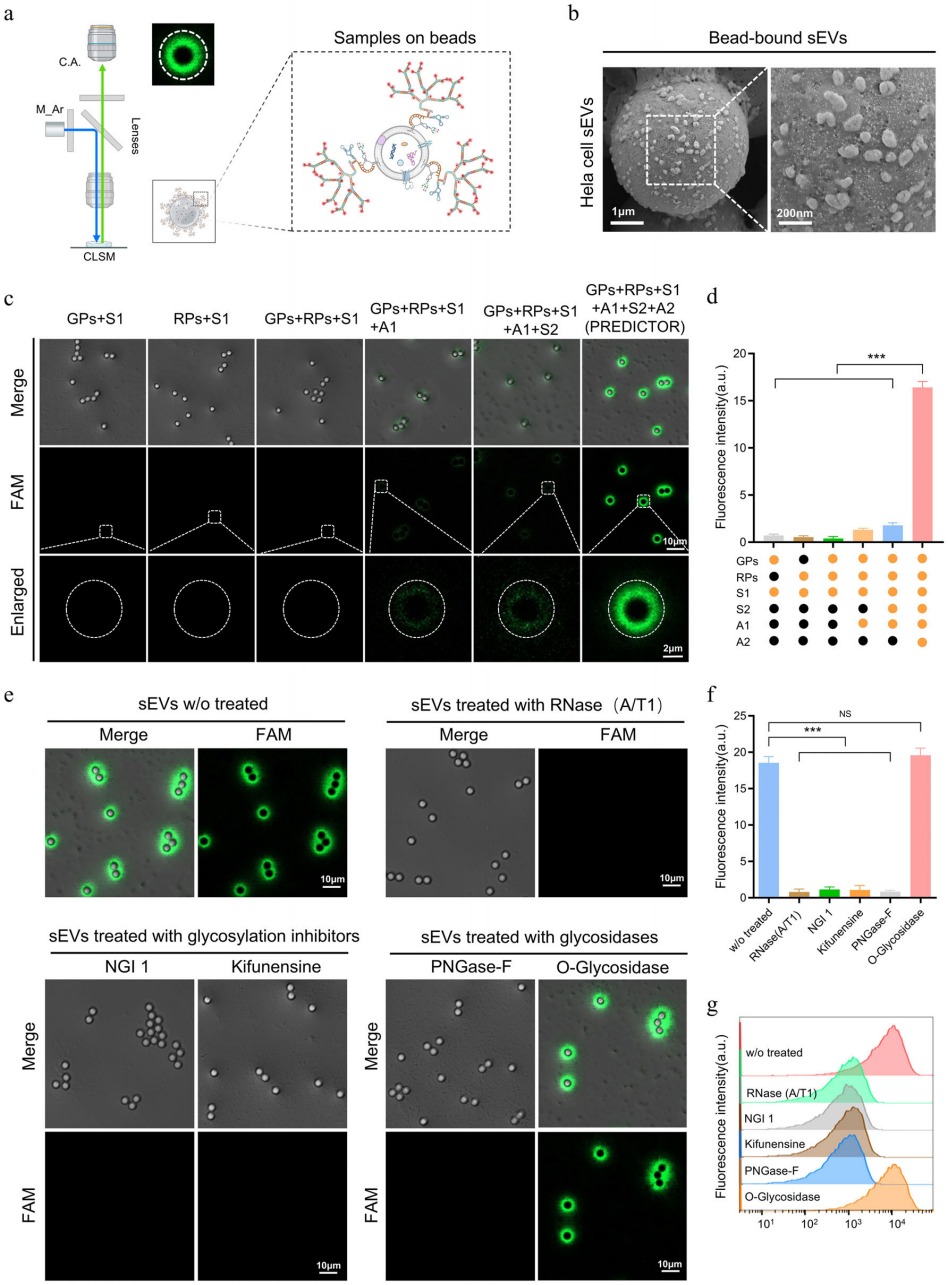
**Specificity validation of PREDICTOR for surface glycoRNAs on sEVs**. (a) Principle of PREDICTOR: dual recognition by the glycan probe (GP; Neu5Ac aptamer) and RNA probe (RP; sequence‐specific hybridization) reconstitutes an initiator that triggers dendritic DNA growth and fluorescence amplification on the sEV surface. (b) SEM image of sEVs captured on latex beads. (c) CLSM images of bead‐captured sEVs processed with PREDICTOR or with the indicated component‐omission controls. Scale bars are indicated. (d) Quantification of mean fluorescence intensity in c (a.u.). (e) CLSM images of HeLa‐derived sEVs pre‐treated with RNase A/T1, kifunensine, or glycosidases (PNGase F, O‐glycosidase) prior to PREDICTOR readout. Scale bars are indicated. (f) Quantification of mean fluorescence intensity in e (a.u.). (g) Flow cytometry analysis of PREDICTOR fluorescence on bead‐captured HeLa‐derived sEVs under the indicated treatments. Data are mean ± SD from three independent experiments. Statistical significance was determined by one‐way ANOVA with Tukey's multiple‐comparison test; ns, not significant; ***p < 0.001.

To visualize surface glycoRNAs at the single‑vesicle level, purified sEVs were first immobilized on aldehyde/sulfate latex beads and subjected to the PREDICTOR reaction directly on the bead‑captured vesicles. Scanning electron microscopy confirmed dense coverage of sEVs on the bead surface (Figure 3b). CLSM revealed strong fluorescence on beads only when all PREDICTOR components were present, while omission of essential components produced minimal background (Figure 3c,d).

To verify that the signal originates from glycoRNAs rather than nonspecific probe adsorption, we independently perturbed the RNA and glycan moieties prior to PREDICTOR readout. Pre‑treatment of intact sEVs with an RNase cocktail (RNase A/T1) markedly reduced fluorescence compared with matched buffer controls (Figure 3e,f), indicating that surface‑accessible RNA is required. Likewise, removal of N‑glycans with PNGase F or cleavage of terminal sialic acids with neuraminidase substantially suppressed the PREDICTOR signal, whereas O‑glycosidase had little effect (Figure 3e,f). Perturbation of N‑glycan maturation with kifunensine (an ER α‑mannosidase I inhibitor) also decreased the signal, consistent with the dependence of our Neu5Ac‑aptamer recognition on sialylated N‑glycan features.

Flow cytometry of bead‑captured sEVs provided an orthogonal readout and corroborated the microscopy results: RNase A/T1, PNGase F, neuraminidase, or kifunensine treatment significantly reduced the mean fluorescence intensity relative to untreated controls (Figure 3g). Together, these data demonstrate that PREDICTOR reports surface‑accessible, sialylated N‑glycan‑linked RNAs on intact sEVs and that the proximity‑gated DNA circuit effectively suppresses background from unbound probes.

To evaluate the multifunctionality of PREDICTOR, it was also applied to MCF‐10A cells to detect cell surface glycoRNAs (Figure S2). MCF‐10A cells were treated with RNase cocktail (A/T1), NGI‐1, and PNGase‐F, followed by CLSM. Furthermore, flow cytometry analysis of the same treated samples confirmed these findings, further validating the conclusions. These results indicate that PREDICTOR can be utilized for in situ imaging of glycoRNAs with high sensitivity and selectivity.

To quantitatively benchmark PREDICTOR against representative glycoRNA amplification workflows, we compared it with ARPLA and HieCo2 under matched conditions using serial sEVs inputs (5×10^3^–5×10^9^ particles·mL^−^^1^). In bulk fluorescence measurements, regression of the fluorescence readout versus log_10_(sEVs concentration) yielded a steeper response for PREDICTOR (*y* = 0.3723*x*‐0.8453, *R* ^2^ = 0.9846) than for ARPLA (*y* = 0.3013*x*‐0.6477, *R* ^2^ = 0.9748) and HieCo2 (*y* = 0.2303*x*‐0.4030, *R* ^2^ = 0.9663), and PREDICTOR produced a higher endpoint signal under identical acquisition settings (Figure S3). In bead‑based imaging, PREDICTOR similarly showed a steeper log_10_ linear response (*y* = 4.1050*x*‐9.4807, *R* ^2^ = 0.9816) than ARPLA (*y* = 3.4951*x*‐8.5349, *R* ^2^ = 0.9748) and HieCo2 (*y* = 2.7110*x*‐5.7566, *R* ^2^ = 0.9757) (Figure S4). These benchmarking data highlight the enhanced signal gain achieved by the enzyme‑free, non‑linear DNA cascade in PREDICTOR.

Lipid rafts are cholesterol‑and sphingolipid‑enriched membrane microdomains that help organize signalling components and glycosylated biomolecules. Previous work has reported co‑localization of cell‑surface glycoRNAs with lipid rafts (Ma et al. 2024). Because sEVs membranes retain raft‑like domains, we next asked whether surface glycoRNAs are spatially associated with lipid rafts on sEVs. Using HeLa‑derived sEVs as a model, ganglioside GM1‑enriched lipid rafts were stained with Alexa Fluor 555‑conjugated cholera toxin subunit B (CT‑B), and glycoRNAs were imaged with PREDICTOR. CLSM revealed pronounced co‑localization of the PREDICTOR signal with CT‑B staining on sEVs (Figure S5a). Quantitative analysis yielded a Pearson correlation coefficient of 0.653 ± 0.145 (Figure S5c). Furthermore, flow cytometry analysis of sEVs treated as described indicated that the double‐stained sEVs predominantly accumulated in the Q2 quadrant, accounting for 37.2%, which further supports the co‐localization of glycoRNA and lipid rafts on sEVs (Figure S5d). Extending this analysis to intact cells, CT‑B staining and PREDICTOR imaging on MCF‑10A membranes similarly showed co‑localized patterns (Pearson correlation coefficient 0.585 ± 0.117; Figure S6). These results illustrate that PREDICTOR can be used not only for sensitive detection but also for spatial mapping of glycoRNAs distribution on nanoscale sEVs and cell surfaces.

### 3.4 Abundance of glycoRNAs in Breast Cancer Transformation

sEVs act as short‑ and long‑range messengers in physiology and disease and have become an important source of non‑invasive biomarkers (Couch et al. 2021; Kalluri and McAndrews 2023; Takahashi and Takakura 2023). Given prior reports linking surface glycoRNAs to malignant phenotypes, we next asked whether PREDICTOR could quantify glycoRNA abundance across a breast‑cancer malignant‑transformation series. We selected three representatives small RNAs previously reported as glycoRNA candidates (U1 snRNA, SNORD2, and U8) and applied PREDICTOR to sEVs derived from MCF‑10A (non‑malignant), MCF‑7 (low‑malignant), and MDA‑MB‑231 (high‑malignant) cells.

**FIGURE 4:**
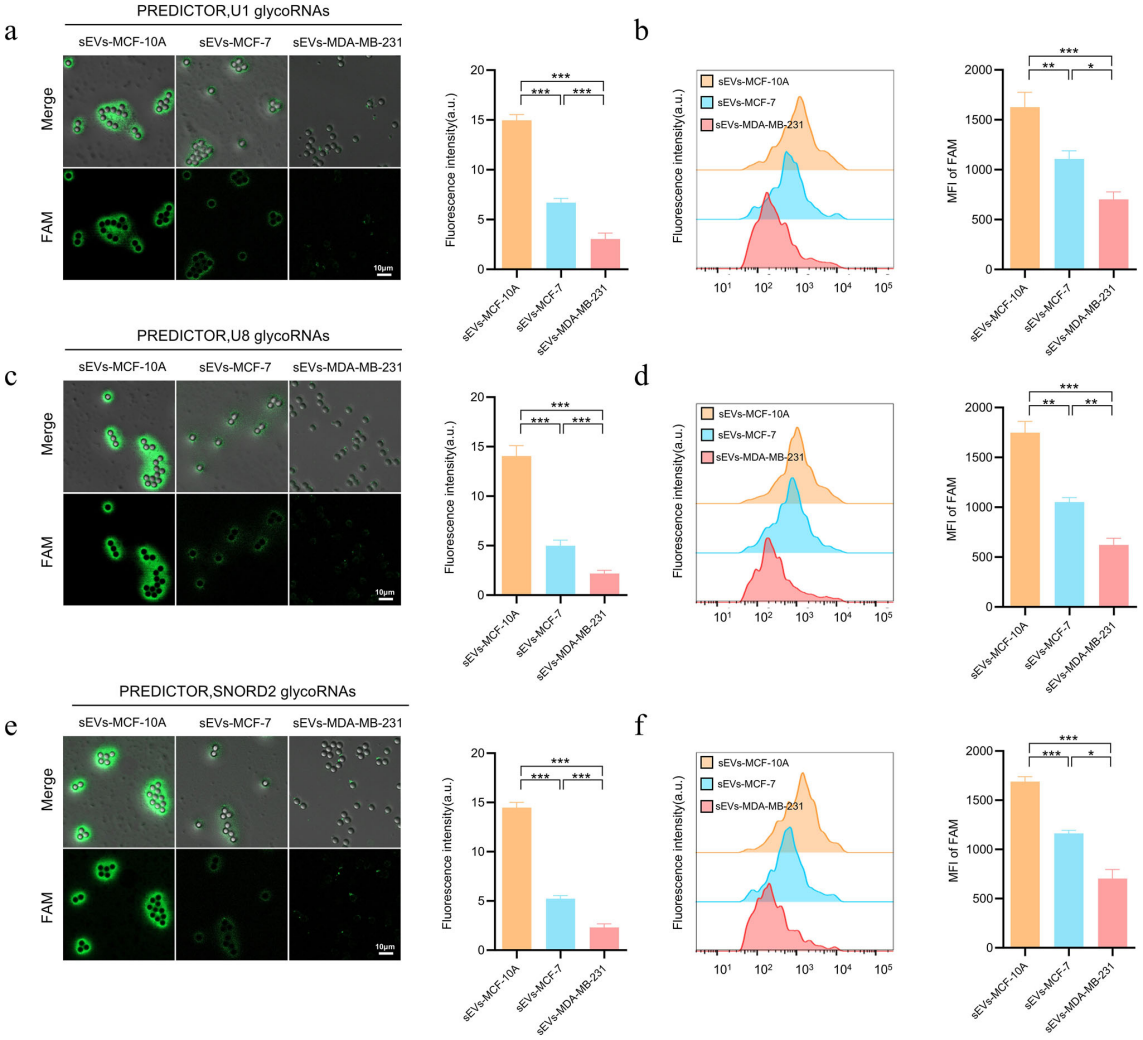
**｜ Profiling surface glycoRNA abundance on sEVs across breast‐cancer malignant transformation using PREDICTOR**. (a) CLSM images of U1, SNORD2, and U8 surface glycoRNAs on sEVs derived from MCF‐10A, MCF‐7, and MDA‐MB‐231 cells. (b) Quantification of mean fluorescence intensity per bead in a (a.u.; *n* = 3). (c) Flow cytometry analysis of PREDICTOR fluorescence for the indicated glycoRNA targets on sEVs. (d) Quantification of mean fluorescence intensity from c (*n* = 3). Data are mean ± SD. Statistical significance was determined by one‐way ANOVA with Tukey's multiple‐comparison test; **p < 0.01, ***p < 0.001.

CLSM imaging revealed readily detectable surface signals for U1, SNORD2, and U8 on sEVs from all three cell lines (Figure 4a). Quantitative fluorescence analysis showed that the abundance of these surface glycoRNAs decreased along the malignancy series (MCF‑10A → MCF‑7 → MDA‑MB‑231) (Figure 4b), consistent with the click‑labelling results in Figure 1g. Flow cytometry provided an independent, population‑level validation of these trends (Figure 4c), supporting a negative association between sEVs surface glycoRNA abundance and breast‑cancer malignancy. Together, these results demonstrate that PREDICTOR can be used to profile specific surface glycoRNA candidates on intact sEVs and to compare glycoRNA levels across biological conditions.

To further explore whether PREDICTOR can report differences in sEV surface glycoRNAs across a breast cancer cell line series, MCF‐10A, MCF‐7, and MDA‐MB‐231 cells were used as models representing distinct phenotypic states. As shown in Figure S7, PREDICTOR signals decreased across this series, consistent with an inverse association between surface‐accessible glycoRNA signal and malignant phenotype in this cell line model.

### 3.5 Surface glycoRNAs Contribute to sEVs Membrane Stiffness

**FIGURE 5:**
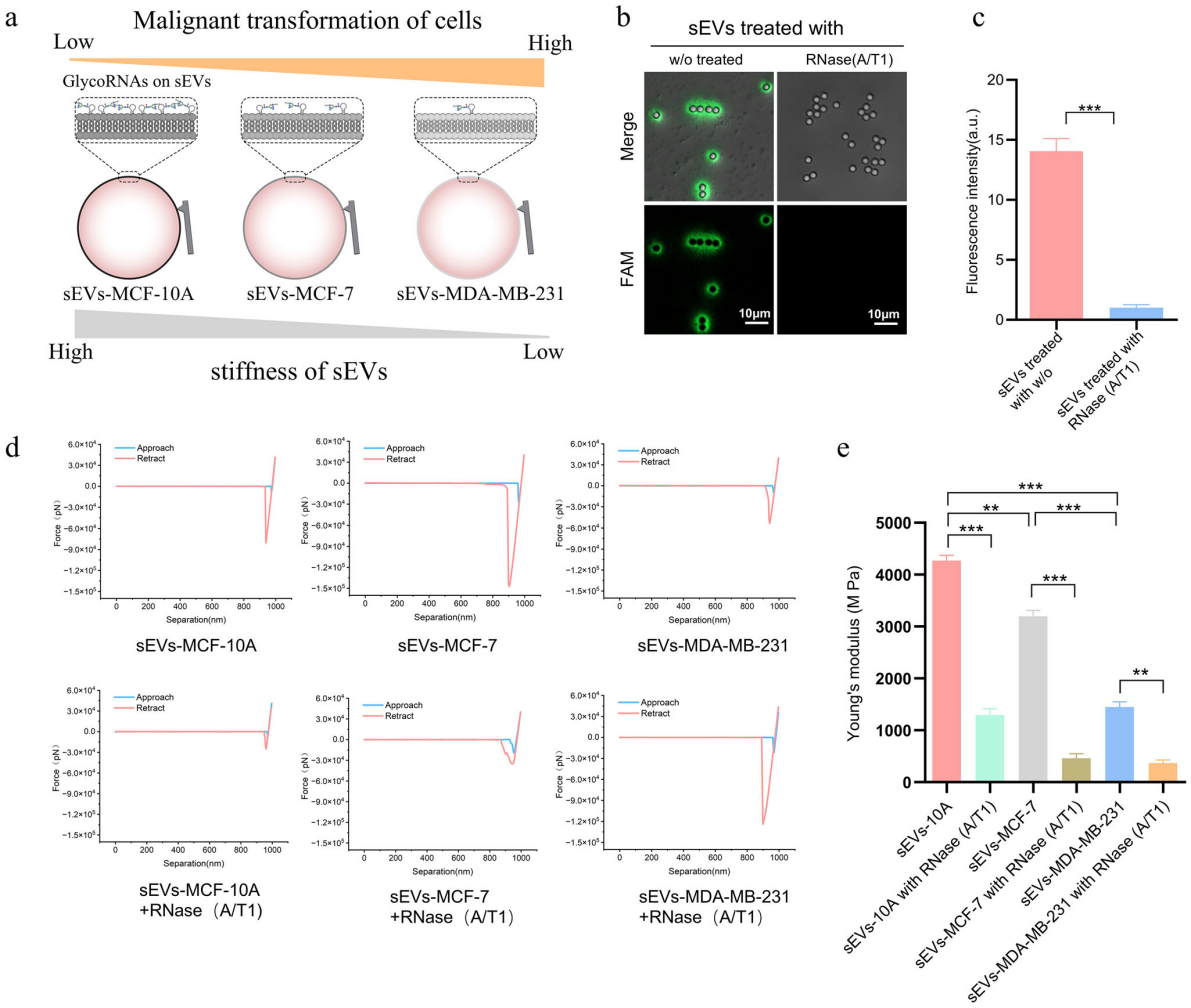
**Surface glycoRNAs correlate with sEV membrane stiffness**. (a) Schematic of the relationship between surface glycoRNA abundance and sEV stiffness. (b) PREDICTOR readout of surface glycoRNAs on sEVs following RNase A/T1 treatment or RNase‐inhibitor protection (*n* = 3). (c) Quantification of mean fluorescence intensity per bead in b (a.u.). (d) Representative AFM force–separation curves fitted with the Hertz model. (e) Young's modulus of sEVs derived from MCF‐10A, MCF‐7, and MDA‐MB‐231 cells with or without RNase A/T1 treatment. Data are mean ± SD. Statistical significance was determined by one‐way ANOVA with Tukey's multiple‐comparison test; **p < 0.01, ***p < 0.001.

To investigate the impact of glycoRNAs levels on the mechanical properties of sEVs and to explore the relationship between the mechanical properties of sEVs and the malignant transformation of breast cancer, we employed Atomic Force Microscopy (AFM) to assess the stiffness of sEVs. Given that the abundance of glycoRNAs decreases with the increasing malignancy of breast cancer cell lines, we measured the stiffness of sEVs derived from cells of varying malignancy (Figure 5a). We utilized the PREDICTOR technology to verify the absence of surface glycoRNAs in sEVs treated with RNase (A/T1) and performed statistical analysis on the PREDICTOR fluorescence intensity (Figure 5b,c). Nanoindentation tests on sEVs using AFM yielded force–displacement curves resulting from the interaction between the probe and the surface of sEVs, with Young's modulus values used to characterize the stiffness of sEVs. The results indicated that the loss of surface glycoRNAs in sEVs derived from the MCF‐10A, MCF‐7, and MDA‐MB‐231 cell lines led to a reduction in surface stiffness. Specifically, as the malignancy of the tumour increased, the surface stiffness of sEVs decreased by 25.08% and 54.72%, respectively (Figure 5d,e). These findings suggest that the downregulation of glycoRNAs on the surface of sEVs contributes to this softening effect, indicating that surface RNA‑associated components can measurably influence sEVs membrane stiffness.

### 3.6 Surface glycoRNAs Modulate Macrophage Uptake and Inflammatory Activation

**FIGURE 6:**
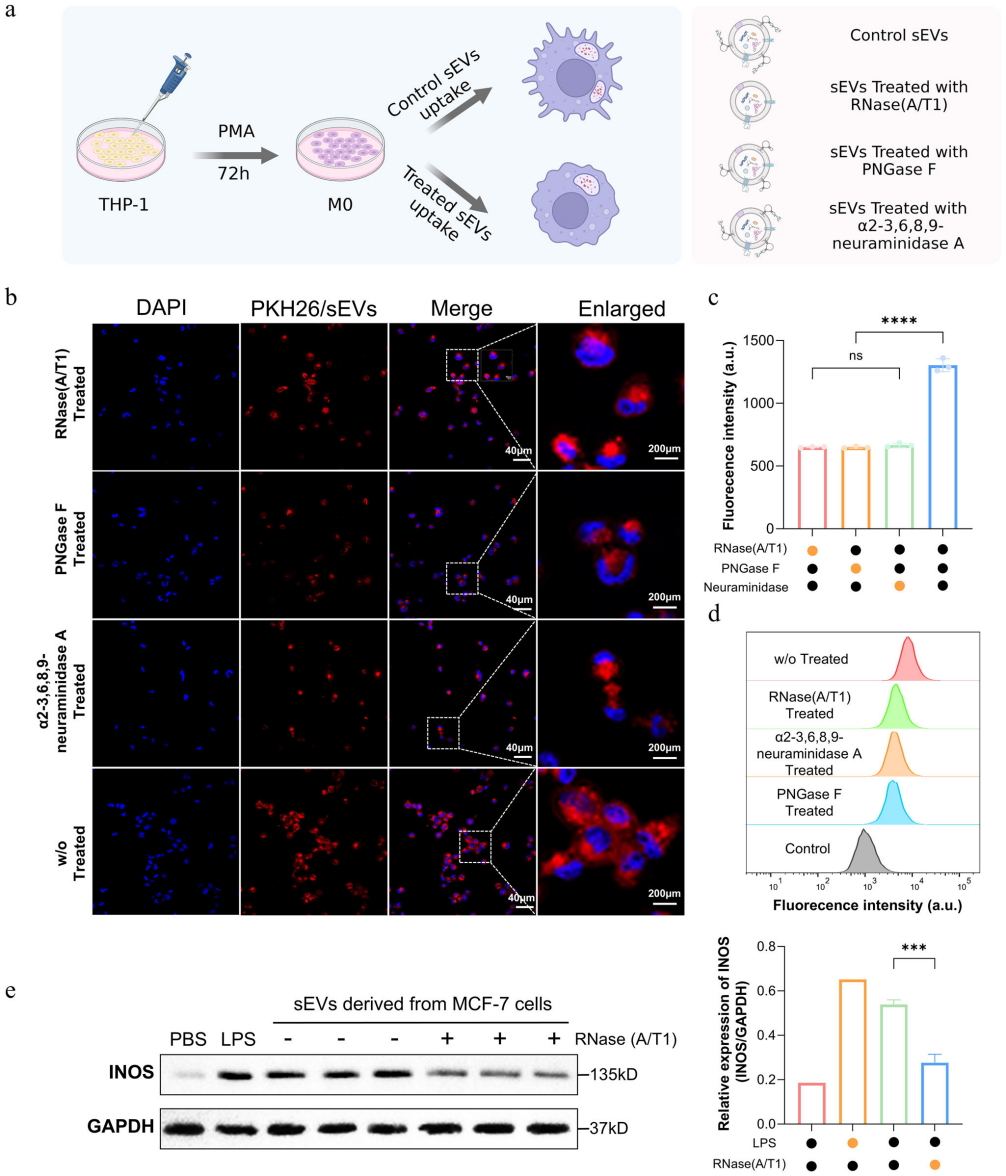
**Syngeneic human macrophage uptake assay using treated versus control sEVs**. (a) Experimental scheme: THP‐1 cells were differentiated into M0 macrophages (PMA, 72 h) and incubated with control or enzymatically treated MCF‐7 sEVs. (b) Confocal images of macrophages incubated with PKH26‐labeled sEVs (red) and nuclei stained with DAPI (blue); merged and enlarged views are shown. Scale bars are as indicated. (c) Quantification of sEV‐associated cellular fluorescence under the indicated treatment conditions (mean ± SD; *n* = 3). (d) Representative flow‐cytometry histograms of cellular fluorescence. (e) Immunoblot analysis of iNOS with GAPDH as a loading control under the indicated treatment conditions; right, densitometric quantification.

To explore whether surface glycoRNAs contribute to immune‑cell interactions with tumour‑derived sEVs, we evaluated the uptake of treated versus control sEVs by macrophages. Because cross‑species assays may confound receptor–ligand interactions, we used a syngeneic human system in which THP‑1 monocytes were differentiated into M0 macrophages and incubated with PKH26‑labeled MCF‑7‑derived sEVs (Figure 6a). Enzymatic depletion of surface RNA (RNase A/T1) or removal of sialylated N‑glycan features (PNGase F or neuraminidase) significantly reduced cellular association/uptake of sEVs as assessed by CLSM and flow cytometry (Figure 6b–d).

To assess whether altered vesicle uptake was accompanied by changes in macrophage activation, we probed inducible nitric‐oxide synthase (iNOS) as a representative pro‐inflammatory marker. Macrophages exposed to control sEVs showed higher iNOS levels than those incubated with glycoRNA‐depleted or desialylated sEVs in the same experimental setting (Figure 6e), indicating that surface glycoRNA/glycan features can modulate macrophage responses.

Motivated by reports that glycoRNAs can serve as ligands for Siglec receptors (Flynn et al. 2021; Graziano et al. 2025; Li et al. 2025), we performed supplementary experiments to probe Siglec involvement in our human macrophage model. Recombinant Siglec‑11 showed detectable binding/recognition signals that were sensitive to RNase (A/T1) treatment of THP‑1‑derived M0 macrophage surface‑accessible RNA (Figure S8). Northern blot analysis of THP‑1 total RNA further supported the RNase sensitivity of the Siglec‑11‑responsive RNA signal (Figure S9). These findings are consistent with a possible contribution of sialylated glycoRNA features to receptor engagement; however, definitive identification of the responsible receptor(s) and uptake pathway(s) will require dedicated mechanistic studies.

## 4 Discussion

Here we developed PREDICTOR, an enzyme‑free, proximity‑encoded catalytic DNA circuit that enables selective in situ imaging and quantification of surface glycoRNAs on intact sEVs. By requiring simultaneous recognition of the glycan (Neu5Ac) and RNA components before initiating a non‑linear HCR cascade, PREDICTOR combines strong background suppression with substantial signal gain. Benchmarking against representative amplification workflows (ARPLA and HieCo2) under matched conditions showed that PREDICTOR provides a steeper concentration‐response and higher endpoint fluorescence across a wide sEVs input range (Figure S3, S4; Table S2). Together with the absence of enzymatic steps, these features make PREDICTOR operationally simple and well suited for low‑abundance targets on nanoscale vesicles.

Complementing existing glycoRNA technologies‐including ARPLA (enzyme‑assisted proximity ligation/rolling‑circle amplification), drFRET (FRET readout on sEVs), HieCo2 (DNA‑circuit amplification), and the recently reported IPIA strategy(Gong et al. 2025; Liu et al. 2024; Ma et al. 2024; Ren et al. 2025), PREDICTOR expands the spectrum of programmable in situ reaction networks that can be deployed for glycoRNA visualization. Importantly, because glycoRNAs have been reported both on vesicle surfaces and within vesicle lumens, the ability to localize the signal to the intact sEV membrane provides a path to dissect surface‑exposed glycoRNA biology separately from luminal glyco‑modified RNA cargo.

Applying PREDICTOR to a breast‑cancer transformation series revealed a consistent decrease in surface glycoRNA abundance with increasing malignancy. This trend, together with the observed co‑localization of glycoRNAs with lipid‑raft domains, supports the notion that surface glycoRNAs may be organized within membrane microdomains rather than randomly distributed. Because membrane microdomains and surface‑associated macromolecules can contribute to vesicle mechanics, our AFM measurements further suggest that surface glycoRNA features measurably influence sEVs membrane stiffness.

Functionally, we found that enzymatic depletion of surface RNA or desialylation reduced THP‐1 macrophage association/uptake of MCF‐7 sEVs and attenuated iNOS induction. These results are consistent with sialylated glycoRNA features contributing to macrophage recognition. glycoRNAs have been reported to bind Siglec receptors (Flynn et al. 2021; Li et al. 2025), and Siglec family members can mediate the binding and internalization of sialylated particles including extracellular vesicles (Schmidt et al. 2023). Our RNase‐sensitive Siglec‐11 binding assay provides supportive evidence for Siglec involvement, but does not exclude contributions from other receptors or uptake routes (e.g., clathrin‐mediated endocytosis, macropinocytosis, and other endocytic pathways, as well as additional sialic‐acid‐binding receptors). Future work combining receptor perturbation and pathway‐inhibitor studies will be needed to establish causal mechanisms.

Several limitations should be acknowledged. First, PREDICTOR was validated here primarily on cultured‑cell‑derived sEVs; its performance in clinical samples and other complex matrices (e.g., serum, plasma, or tissue interstitial fluid) will require further optimization of sEVs enrichment, blocking/washing, and background control. Second, because the assay is performed on intact, non‑permeabilized vesicles, PREDICTOR reports surface‑accessible glycoRNAs and is not expected to detect luminal glyco‑modified RNAs without additional permeabilization steps. Third, the RNA‑probe component targets predefined sequences; therefore, unbiased discovery of unknown glycoRNA sequences will require integration with orthogonal enrichment/sequencing workflows. Finally, the current glycan probe uses a Neu5Ac‑binding aptamer; expanding probe repertoires to additional glycan epitopes and enabling multiplexed readouts should further broaden the applicability of this platform.

## Author Contributions

**Shuang Xie**: methodology, investigation, visualization, writing – original draft, writing – review and editing, data curation. **Ben Niu**: investigation, writing – original draft, writing – review and editing. **Ruijia Deng**: writing – original draft, writing – review and editing. **Liu Feng**: methodology, investigation. **Zuowei Xie**: investigation. **Shuang Zhao**: methodology, resources. **Hongzhao Yang**: methodology, investigation. **Meilin Gong**: methodology, visualization. **Jing Sheng**: methodology, visualization. **Ligai Zhang**: methodology, visualization. **Yan Pi**: methodology, investigation, writing – review and editing, supervision, conceptualization. **Ningtao Cheng**: methodology, investigation, writing – review and editing, supervision, conceptualization. **Ming Chen**: conceptualization, investigation, writing – review and editing, methodology, writing – original draft. **Kai Chang**: methodology, investigation, writing – review and editing, writing – original draft, supervision, funding acquisition, validation.

## Ethics Statement

Not applicable. This study used established cell lines and did not involve human participants or animal experiments.

## Conflicts of Interest

The authors declare no conflicts of interest.

## Supporting information



Supporting material: jev270282‐Sup‐0001‐TableS1.docx

Supporting material: jev270282‐Sup‐0002‐TableS2.docx

Supporting material: jev270282‐Sup‐0003‐SuppMat.docx

## Data Availability

The data that support the findings of this study are available from the corresponding author upon reasonable request.
