# Supplementary material for: PREDICTOR: A Non‐Enzymatic Catalytic Cascade Tool for in Situ Visualization of Small Extracellular Vesicle Surface glycoRNAs
Source: J Extracell Vesicles. 2026 Apr 14;15(4):e70282. doi: 10.1002/jev2.70282 (PMC13077550; doi:10.1002/jev2.70282)
Supplement: Supplementary file 1 — Supporting material: jev270282‐Sup‐0001‐TableS1.docx [file JEV2-15-e70282-s001.docx]

Table S1. DNA sequences and modifications

| Oligonucleotide names | Sequence (5’→3’) |
| --- | --- |
| Trigger | TGACGAACTAGTTGATGAAGCTG |
| F-strand of substrate-1 | FAM_GTGTGCCTATTATGTCTCCTCCTGTGTGCCTATTATGTCTCCTCCTCAGCTTCATCAACTAGTTCGTCA |
| Q-strand of substrate-1 | AACTAGTTGATGAAGCTGGACATAATAGGCACACGACATAATAGGCACAC_BHQ1 |
| Assistant-1 | GTGCCTATTATGTCGTGTGCCTATTATGTCCAGCTT |
| Assistant-2 | GCACACCTAGTTGATGAAGC |
| F-strand of substrate-2 | AGGAGGAGACATAATAGGCATGACGAACTAGTTGATGAAGCTG_FAM |
| Q-strand of substrate-2 | BHQ1_CAGCTTCATCAACTAGTGCCTATTATGTCTC |
| Glycan probe | TGACGAACTAGTTGATATGACATTTTTTTTTTTTTTTTTTTTTTTTTTTTTTAGGGAATTCGTCGACGGATCCCGTGGCGTCTGCAACGGAAAAGAATTTATCTTGTCCTGCAGGTCGACGCATGCGCCG |
| RNA probe-U1 | CTGGGAAAACCACCTTCGTGATCATGGTATCTCCCCTGCCAGGTAAGTATTTTTTTTTTTTTTTTTTTTTTTTTTTTTTTTGTCATATGAAGCTG |
| RNA probe-SNORD2 | CAGGTCAGTCCCGAAAGATGATTGCCATCATTTCTTTTTTTTTTTTTTTTTTTTTTTTTTTTTTTGTCATATGAAGCTG |
| RNA probe-U1 | CTGGGAAAACCACCTTCGTGATCATGGTATCTCCCCTGCCAGGTAAGTATTTTTTTTTTTTTTTTTTTTTTTTTTTTTTTTGTCATATGAAGCTG |
| RNA probe-U8 | TAATCTGCCCTCCGGAGGAGGAACAGGTAAGGATTATTTTTTTTTTTTTTTTTTTTTTTTTTTTTTTGTCATATGAAGCTG |
|  |  |
