# Supplementary material for: PREDICTOR: A Non‐Enzymatic Catalytic Cascade Tool for in Situ Visualization of Small Extracellular Vesicle Surface glycoRNAs
Source: J Extracell Vesicles. 2026 Apr 14;15(4):e70282. doi: 10.1002/jev2.70282 (PMC13077550; doi:10.1002/jev2.70282)
Supplement: Supplementary file 2 — Supporting material: jev270282‐Sup‐0002‐TableS2.docx [file JEV2-15-e70282-s002.docx]

**Table S2 Comparison of PREDICTOR with representative glycoRNA assays**

| **Method** | **Principle** | **Signal**  **Amplification** | **enzymatic reactions** | **Main Advantages** | **Limitations** | **Time** | **Ref** |
| --- | --- | --- | --- | --- | --- | --- | --- |
| **ARPLA** | Sialic acid aptamers and specific DNA probes enable dual recognition, and rolling circle amplification (RCA) enables signal amplification | Linear amplification | Yes | Spatial imaging in single cells | Signal intensity is relatively weak | 4h | 1 |
| **drFRET** | Sialic acid aptamers and specific DNA probes enable dual recognition, and FRET is utilized | No amplification | No | In situ detection of glycoRNA on sEVs | Signal intensity is relatively weak | 70min | 2 |
| **HieCo2** | Incorporation of MCRs is followed by dual recognition, and hybridization chain reaction (HCR) enables signal amplification | Linear amplification | No | Quantification of glycosylation sites | Complex operation, and long time required | 3h | 3 |
| **IPIA** | Sialic acid aptamers and specific DNA probes enable dual recognition, and HCR-triggered G_4_ formation is utilized | Linear amplification | No | Spatial imaging of glycoRNA in living cells and zebrafish | Signal intensity is relatively weak | 2.3h | 4 |
| **PREDICTOR** | Sialic acid aptamers and specific DNA probes enable dual recognition, and non-linear HCR enables signal amplification | Non-linear amplification | No | Fast and simple; sensitive detection of glycoRNAs on sEVs and cells | Intracellular glycoRNA cannot be visualized | 1h | This work |
