# Supplementary material for: PREDICTOR: A Non‐Enzymatic Catalytic Cascade Tool for in Situ Visualization of Small Extracellular Vesicle Surface glycoRNAs
Source: J Extracell Vesicles. 2026 Apr 14;15(4):e70282. doi: 10.1002/jev2.70282 (PMC13077550; doi:10.1002/jev2.70282)
Supplement: Supplementary file 3 — Supporting material: jev270282‐Sup‐0003‐SuppMat.docx [file JEV2-15-e70282-s003.docx]

**PREDICTOR: A Non-Enzymatic Catalytic Cascade Tool for In Situ Visualization of Small Extracellular Vesicle Surface GlycoRNAs**

Shuang Xie^1^｜Ben Niu^1^｜ Ruijia Deng^1^｜ Liu Feng^1^｜ Zuowei Xie^1^｜ Shuang Zhao^1^｜ Hongzhao Yang^1^｜ Meilin Gong^1^｜ Jing Sheng^1^｜ Ligai Zhang^1^｜ Yan Pi^2^｜ Ningtao Cheng^3^｜ Ming Chen^1^｜ Kai Chang^1,4^

^1^Department of Clinical Laboratory Medicine, Southwest Hospital, Third Military Medical University (Army Medical University), 30 Gaotanyan, Shapingba, Chongqing 400038, China｜^2^Department of Rehabilitation Medicine, The First Affiliated Hospital of Chongqing Medical University, Chongqing 400042, China｜^3^School of Public Health, Zhejiang University School of Medicine, Hangzhou, Zhejiang 310058, China ｜^4^State Key Laboratory of Trauma and Chemical Poisoning, Army Medical University, 30 Gaotanyan, Shapingba District, Chongqing 400038, China.

**Shuang Xie, Ben Niu and Ruijia Deng contributed equally to this article.**

**Correspondence:**Yan Pi(piyan861105@sina.com)｜Ningtao Chen(ncheng@zju.edu.cn)｜ MingChen(chenming1971@tmmu.edu.cn) ｜ Kai Chang(changkai0203@tmmu.edu.cn)

**Keywords:**glycoRNAs｜small extracellular vesicles｜In situ visualization｜DNA nanotechnology｜Non-enzymatic cascade amplification

Table S1. DNA sequences and modifications

| Oligonucleotide names | Sequence (5’→3’) |
| --- | --- |
| Trigger | TGACGAACTAGTTGATGAAGCTG |
| F-strand of substrate-1 | FAM_GTGTGCCTATTATGTCTCCTCCTGTGTGCCTATTATGTCTCCTCCTCAGCTTCATCAACTAGTTCGTCA |
| Q-strand of substrate-1 | AACTAGTTGATGAAGCTGGACATAATAGGCACACGACATAATAGGCACAC_BHQ1 |
| Assistant-1 | GTGCCTATTATGTCGTGTGCCTATTATGTCCAGCTT |
| Assistant-2 | GCACACCTAGTTGATGAAGC |
| F-strand of substrate-2 | AGGAGGAGACATAATAGGCATGACGAACTAGTTGATGAAGCTG_FAM |
| Q-strand of substrate-2 | BHQ1_CAGCTTCATCAACTAGTGCCTATTATGTCTC |
| Glycan probe | TGACGAACTAGTTGATATGACATTTTTTTTTTTTTTTTTTTTTTTTTTTTTTAGGGAATTCGTCGACGGATCCCGTGGCGTCTGCAACGGAAAAGAATTTATCTTGTCCTGCAGGTCGACGCATGCGCCG |
| RNA probe-U1 | CTGGGAAAACCACCTTCGTGATCATGGTATCTCCCCTGCCAGGTAAGTATTTTTTTTTTTTTTTTTTTTTTTTTTTTTTTTGTCATATGAAGCTG |
| RNA probe-SNORD2 | CAGGTCAGTCCCGAAAGATGATTGCCATCATTTCTTTTTTTTTTTTTTTTTTTTTTTTTTTTTTTGTCATATGAAGCTG |
| RNA probe-U1 | CTGGGAAAACCACCTTCGTGATCATGGTATCTCCCCTGCCAGGTAAGTATTTTTTTTTTTTTTTTTTTTTTTTTTTTTTTTGTCATATGAAGCTG |
| RNA probe-U8 | TAATCTGCCCTCCGGAGGAGGAACAGGTAAGGATTATTTTTTTTTTTTTTTTTTTTTTTTTTTTTTTGTCATATGAAGCTG |
|  |  |

**Table S2 Comparison of PREDICTOR with representative glycoRNA assays**

| **Method** | **Principle** | **Signal**  **Amplification** | **enzymatic reactions** | **Main Advantages** | **Limitations** | **Time** | **Ref** |
| --- | --- | --- | --- | --- | --- | --- | --- |
| **ARPLA** | Sialic acid aptamers and specific DNA probes enable dual recognition, and rolling circle amplification (RCA) enables signal amplification | Linear amplification | Yes | Spatial imaging in single cells | Signal intensity is relatively weak | 4h | 1 |
| **drFRET** | Sialic acid aptamers and specific DNA probes enable dual recognition, and FRET is utilized | No amplification | No | In situ detection of glycoRNA on sEVs | Signal intensity is relatively weak | 70min | 2 |
| **HieCo2** | Incorporation of MCRs is followed by dual recognition, and hybridization chain reaction (HCR) enables signal amplification | Linear amplification | No | Quantification of glycosylation sites | Complex operation, and long time required | 3h | 3 |
| **IPIA** | Sialic acid aptamers and specific DNA probes enable dual recognition, and HCR-triggered G_4_ formation is utilized | Linear amplification | No | Spatial imaging of glycoRNA in living cells and zebrafish | Signal intensity is relatively weak | 2.3h | 4 |
| **PREDICTOR** | Sialic acid aptamers and specific DNA probes enable dual recognition, and non-linear HCR enables signal amplification | Non-linear amplification | No | Fast and simple; sensitive detection of glycoRNAs on sEVs and cells | Intracellular glycoRNA cannot be visualized | 1h | This work |


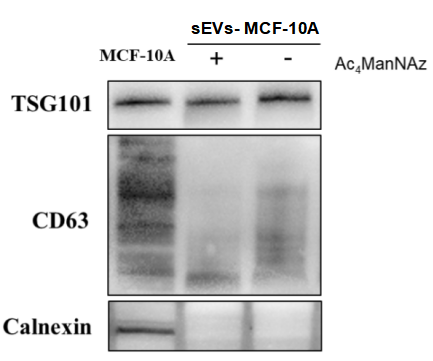


**Figure S1.** **Western blot analysis of the characteristic proteins in cell lysates and sEVs, including TSG101, CD63, and calnexin.**


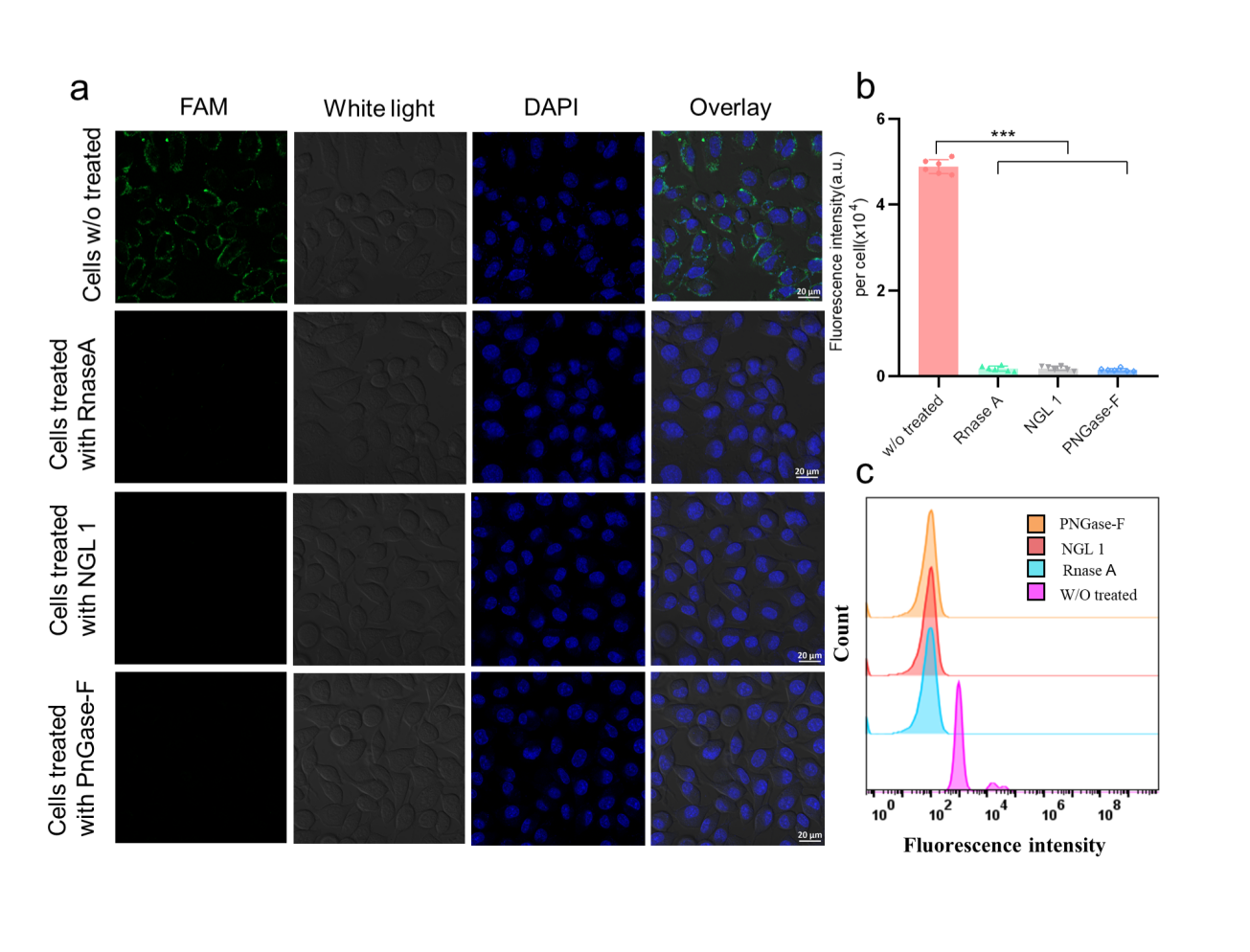


**Figure S2. Validation of PREDICTOR specificity in MCF-10A cells.** (a) CLSM images of surface glycoRNAs detected by PREDICTOR in MCF-10A cells. Cells were pretreated with RNase (A/T1), glycosylation inhibitors, or glycosidases, then fixed and analyzed using PREDICTOR. (b) Quantification of the average fluorescence intensity (AU) per cell from panel a. (c) Flow cytometry analysis of surface glycoRNA fluorescence intensity on MCF-10A cells. Data in panels b and c represent three independent experiments.


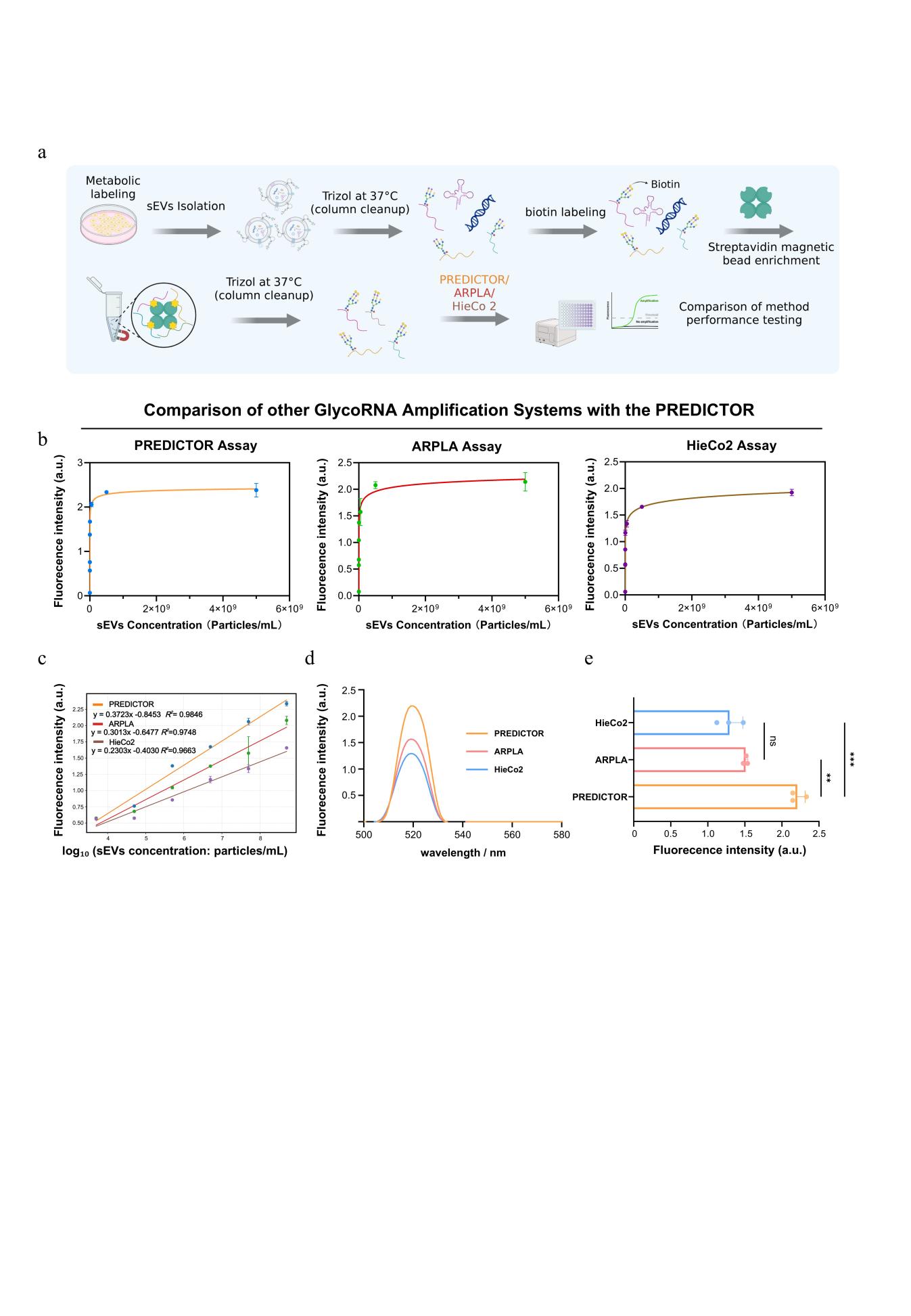


**Figure S3. Quantitative performance benchmarking of PREDICTOR versus representative glycoRNA assays.** (a) Workflow schematic for isolating sEVs, extracting total glycoRNA, and performing method-matched detection for PREDICTOR and comparator assays. (b) Concentration-response curves for PREDICTOR, ARPLA, and HieCo2 under the indicated conditions. (c) Linearized fitting in log_10_ space to compare signal gain across assays. (d) Representative fluorescence emission spectra of the amplified products. (e) Endpoint fluorescence comparison across assays (mean ± SD; n = 3 independent experiments; one-way ANOVA with multiple-comparisons test; ns, not significant; ***P* < 0.01; ****P* < 0.001; ).


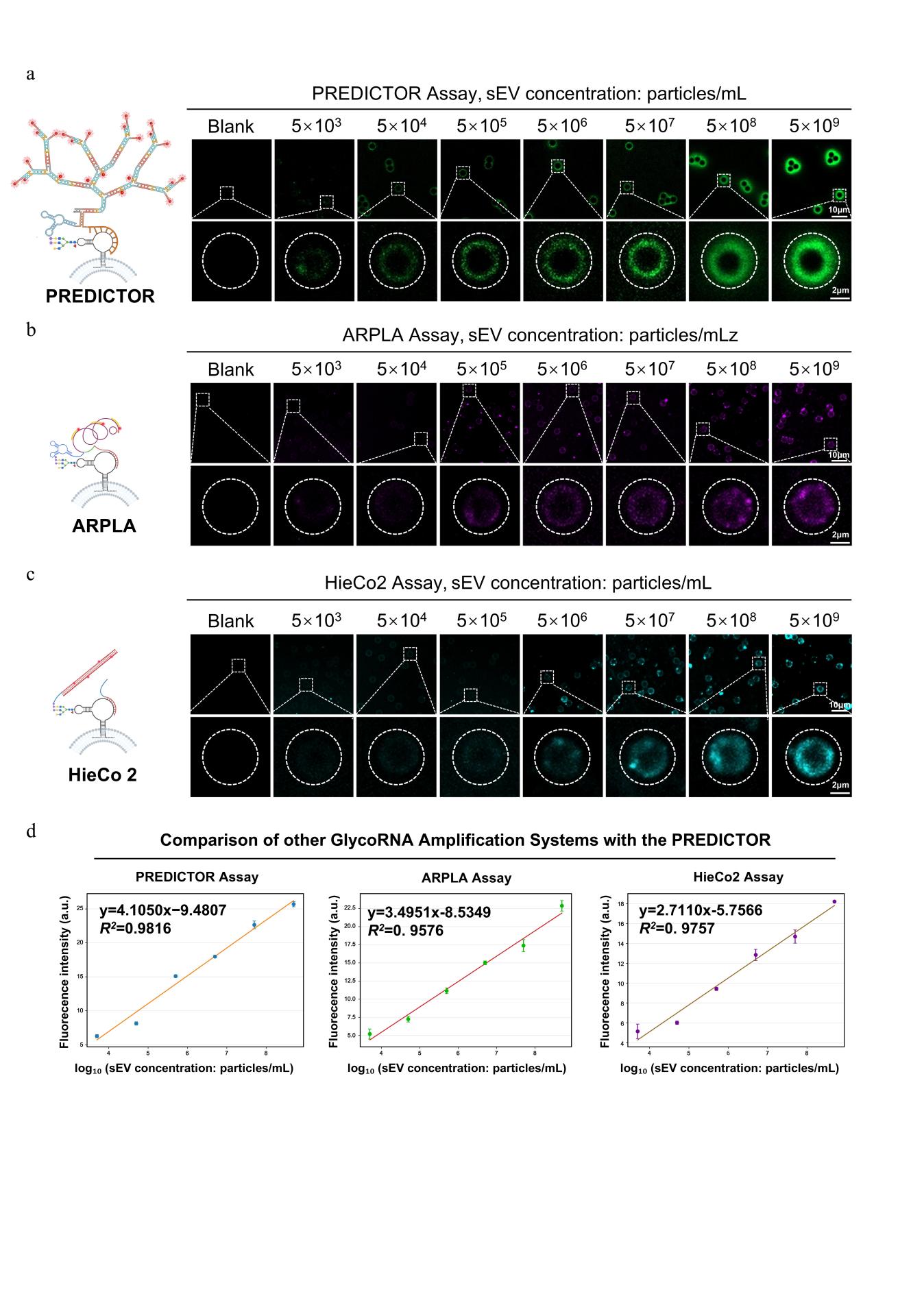


**Figure S4. Bead-based imaging and standard-curve comparison across sEV concentrations.** (a-c) Representative confocal images for PREDICTOR, ARPLA, and HieCo2 performed on serially diluted sEVs (5×10^3^ to 5×10^9^ particles·mL^-^¹). Scale bars are as indicated. (d) Corresponding calibration plots of fluorescence intensity versus log_10_(sEVs concentration), with linear regression equations and R^2^ values.


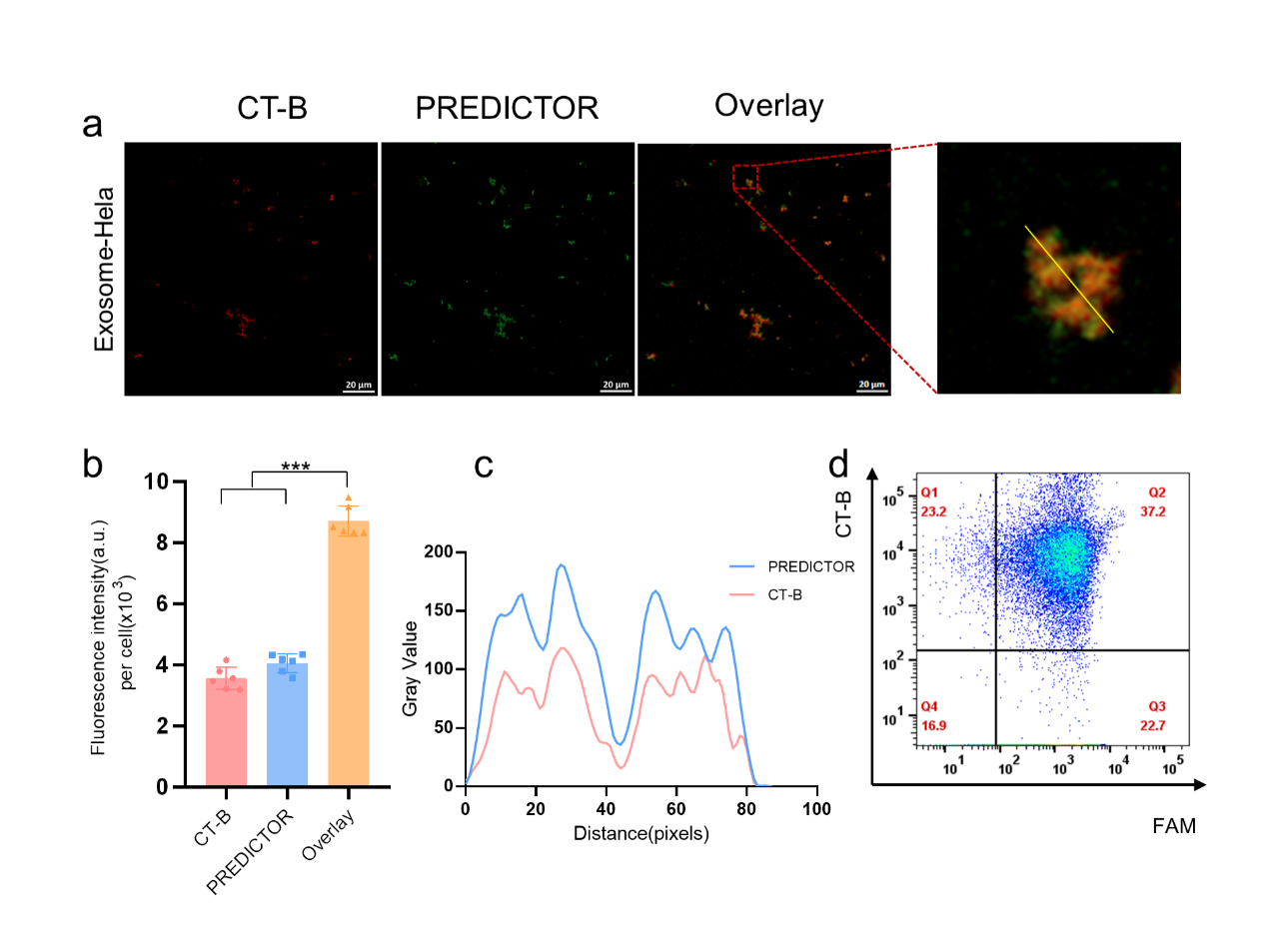


**Figure S5. Spatial distribution of glycoRNAs revealed by PREDICTOR in Exo-HeLa cells. (a**) Representative fluorescence images of lipid rafts, marked by Alexa Fluor 555-labeled CT-B (orange), and glycoRNAs (green), detected using the PREDICTOR method with a U1 RNA probe. (**b**) Quantification of the average fluorescence intensity (AU) per exosome, as shown in panel a. (**c**) Co-localization analysis of lipid rafts and exosomal surface glycoRNAs using Fiji (ImageJ). (**d**) Co-localization of lipid rafts and exosomal surface glycoRNAs as analyzed by flow cytometry.


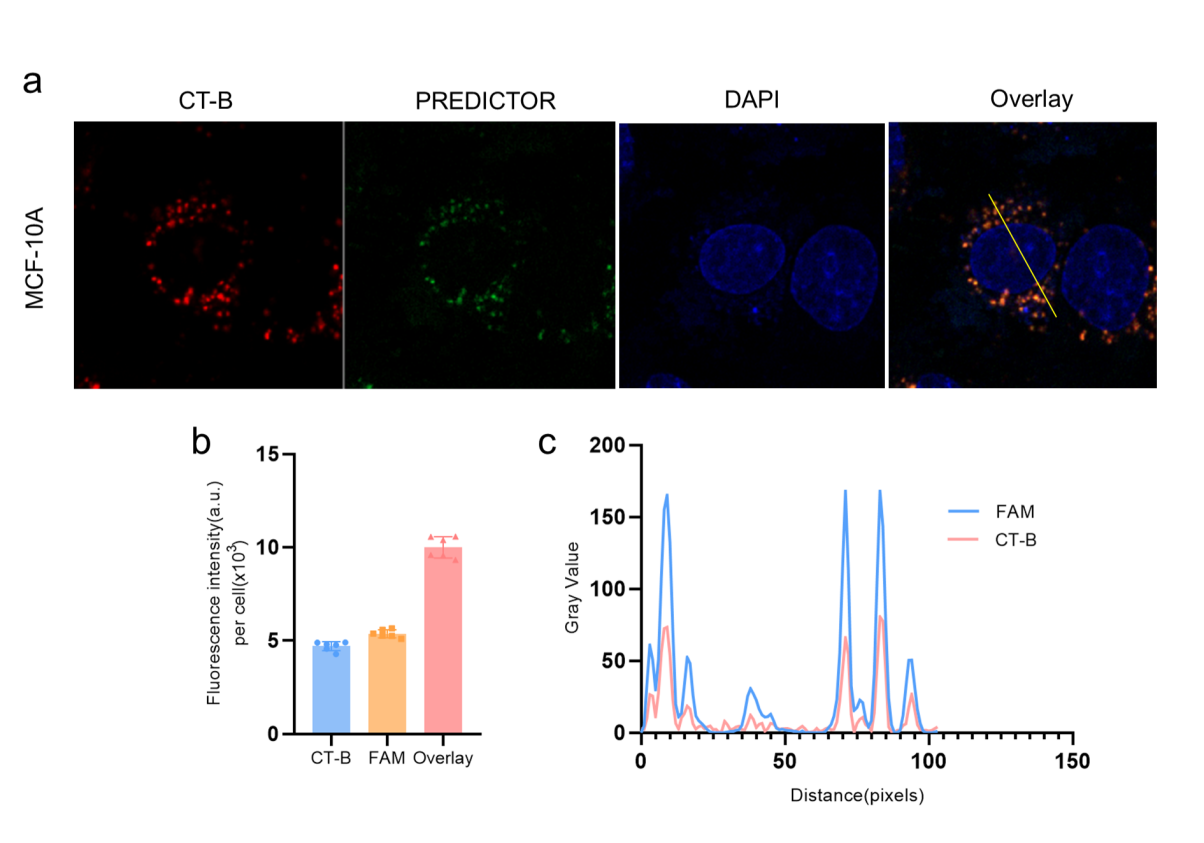


**Figure S6.** **Spatial distribution of glycoRNAs revealed by PREDICTOR in MCF-10A cells**.

1. Representative fluorescence image showing lipid rafts, labeled with Alexa Fluor 555-conjugated CT-B (orange), and glycoRNAs (green), detected using PREDICTOR with the U1 RNA probe. (**b**) Quantification of the average fluorescence intensity (AU) per cell shown in panel a. (**c**) Co-localization analysis of lipid rafts and surface glycoRNAs performed using Fiji (ImageJ).


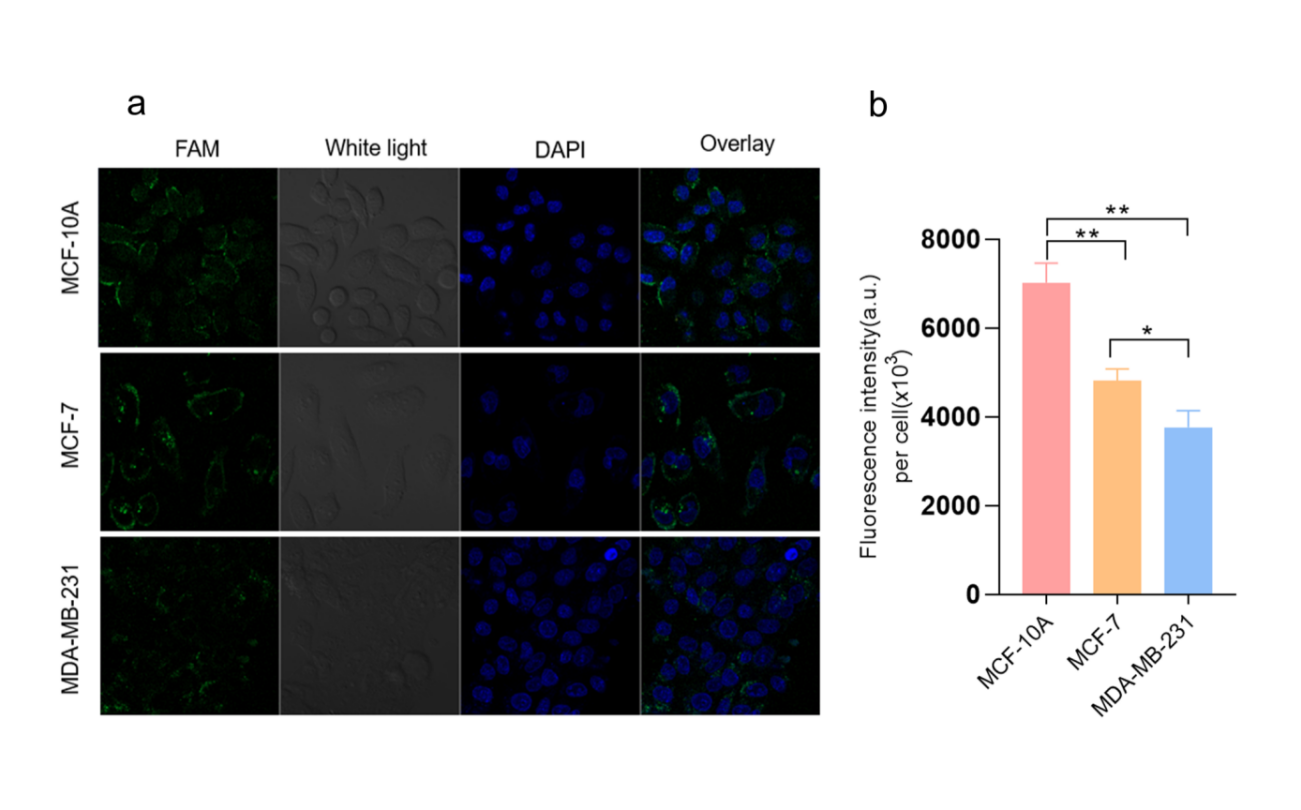


**Figure S7.** **Detection of glycoRNAs abundance on the cellular surface during malignant transformation using PREDICTOR**. (**a)** CLSM images showing the detection of glycoRNAs on the cellular surface using the U1 RNA probe and PREDICTOR in MCF-10A, MCF-7, and MDA-MB-231 cells. The experiment was performed in triplicate. (**b**) Quantification of average fluorescence intensity (AU), as shown in panel a.


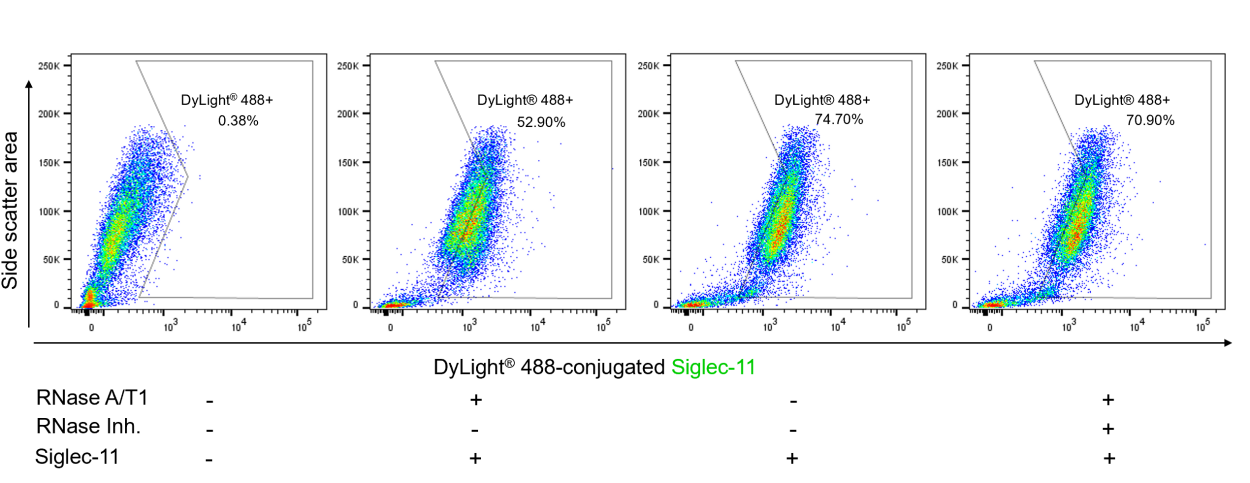


**Figure S8. RNase(A/T1) sensitivity of Siglec-11 interaction with THP-1 derived M0 macrophage surface RNA.** Representative readout for Siglec-11 binding/recognition is shown for control versus RNase(A/T1)‑treated samples.


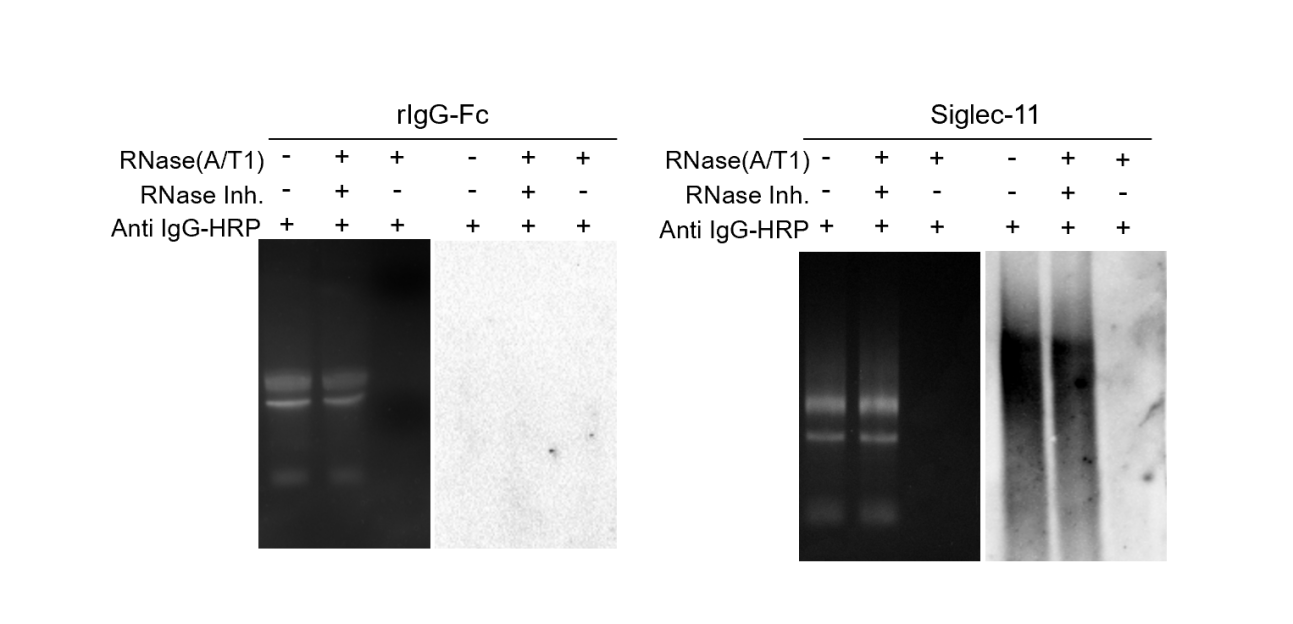


**Figure S9. Northern blot supporting the Siglec-11 interaction assay.** THP-1 derived M0 macrophage total RNA was evaluated with and without RNase(A/T1) treatment to demonstrate RNase sensitivity of the Siglec-11 responsive RNA signal.

**References**

1. Ma Y, Zhang X, Li Q, et al. (2024) Amplification-assisted proximity ligation assay for in situ visualization and quantification of surface glycoRNAs. Nature Biotechnology 42(3): 458–467. https://doi.org/10.1038/s41587-023-02081-y

2. Ren T, He J, Zheng Y, et al. (2025) Tracking glycoRNA on small extracellular vesicles by donor–acceptor fluorescence resonance energy transfer enables sensitive cancer diagnostics. Nature Communications 16(1): 629. https://doi.org/10.1038/s41467-025-55995-4

3. Liu H, Wang H, Chen Z, et al. (2024) In Situ Visualization of RNA-Specific Sialylation in Extracellular Vesicles via Signal Amplification Strategies. Journal of the American Chemical Society 146(47): 32318–32327. https://doi.org/10.1021/jacs.4c09118

4. Gong Z, Feng L, Xie Z, et al. (2025) Intramolecular Proximity-Induced Amplification for Highly Efficient Imaging and Quantification of Surface GlycoRNAs on Small Extracellular Vesicles. Analytical Chemistry 97(41): 17862–17870. https://doi.org/10.1021/acs.analchem.5c02615
